# *In Vivo* Emergence of HIV-1 Highly Sensitive to Neutralizing Antibodies

**DOI:** 10.1371/journal.pone.0023961

**Published:** 2011-08-24

**Authors:** Marlén M. I. Aasa-Chapman, Kelly M. Cheney, Stéphane Hué, Anna Forsman, Stephen O'Farrell, Pierre Pellegrino, Ian Williams, Áine McKnight

**Affiliations:** 1 MRC/UCL Centre for Medical Molecular Virology, Division of Infection and Immunity, University College London, London, United Kingdom; 2 Centre for Immunology and Infectious Disease, Blizard Institute, Queen Mary School of Medicine and Dentistry, London, United Kingdom; 3 Centre for Sexual Health and HIV Research, University College London, London, United Kingdom; Agency for Science, Technology and Research - Singapore Immunology Network, Singapore

## Abstract

**Background:**

The rapid and continual viral escape from neutralizing antibodies is well documented in HIV-1 infection. Here we report *in vivo* emergence of viruses with heightened sensitivity to neutralizing antibodies, sometimes paralleling the development of neutralization escape.

**Methodology/Principal Findings:**

Sequential viral *envs* were amplified from seven HIV-1 infected men monitored from seroconversion up to 5 years after infection. *Env*-recombinant infectious molecular clones were generated and tested for coreceptor use, macrophage tropism and neutralization sensitivity to homologous and heterologous serum, soluble CD4 and monoclonal antibodies IgG1b12, 2G12 and 17b. We found that HIV-1 evolves sensitivity to contemporaneous neutralizing antibodies during infection. Neutralization sensitive viruses grow out even when potent autologous neutralizing antibodies are present in patient serum. Increased sensitivity to neutralization was associated with susceptibility of the CD4 binding site or epitopes induced after CD4 binding, and mediated by complex envelope determinants including V3 and V4 residues. The development of neutralization sensitive viruses occurred without clinical progression, coreceptor switch or change in tropism for primary macrophages.

**Conclusions:**

We propose that an interplay of selective forces for greater virus replication efficiency without the need to resist neutralizing antibodies in a compartment protected from immune surveillance may explain the temporal course described here for the *in vivo* emergence of HIV-1 isolates with high sensitivity to neutralizing antibodies.

## Introduction

In the course of HIV infection the scenario of development of neutralizing antibodies (Nabs) followed by viral escape is well documented. The evolution of viruses to become sensitive to neutralization *in vivo* is counterintuitive, since a growth advantage to a virus that is susceptible to Nabs is difficult to invoke. Here we characterised the sequential development of neutralization sensitive viruses *in vivo* as well as the dynamics of the neutralization escape in the infected individuals.

HIV's envelope (Env) protein is the target for Nabs. A trimeric unit of the surface protein, gp120, and the transmembrane protein, gp41, mediates viral entry into target cells through binding to CD4 and a coreceptor, usually either CCR5 or CXCR4 [1], [2]. After the initial CD4 binding a conformational rearrangement of gp120 variable loops 2 and 3 (V2, V3) results in exposure of the coreceptor binding domain. Concomitantly, or subsequent, conformational changes in gp41 result in gp41-mediated fusion of the viral and plasma membranes. Nabs probably interrupt these processes, with *in vivo* targets being defined through isolation of neutralizing monoclonal antibodies (NMAbs) from infected humans. Broadly NMAbs, or those that neutralize isolates other than the infecting strain, have been described. HK20, 2F5 and 4E10 are directed to the membrane proximal region of gp41 [3], [4], [5], [6]. IgG1b12 and HJ16 recognise different epitopes within the CD4-binding region in gp120 [6], [7], [8]. 2G12 binds a glycan cluster on gp120 [9]. Another group of broadly NMAbs, including 17b, bind epitopes in the coreceptor-binding region which typically is only, or better, exposed following CD4-ligation [10]. HGN194 binds to a conserved epitope in the V3 crown [6]. Two broadly NMAbs, PG9 and PG16, have also been described that preferentially recognise the trimeric Env protein; their epitope recognition relies on V2, V3 and CD4 binding site [11].

Antibodies that neutralize HIV arise with variable rates and potency in infected individuals [12], [13], [14], [15], [16], [17]. Initially, Nabs against the infecting (autologous) virus develop. There is some evidence that autologous targets include linear epitopes in the variable V1/V2 and V3 regions [18], [19], [20], and in the alpha-2 helix of C3 in subtype C infection [20]. Nabs that also can neutralize virus from other patients (heterologous isolates) follows [12], [14], [15], [16], [17]. This response is initially confined to a subset of strains, but can broaden to include geographically diverse virus strains [13]. It is not known whether the heterologous response is the result of maturation of single antibody specificity or the development of antibodies that recognise distinct epitopes in a polyclonal response to one or more epitopes. HIV evolves rapidly in response to Nabs, thus sera rarely neutralize contemporaneous virus efficiently. Indeed, responses against contemporary autologous virus (virus from the same time-point as the serum) are generally undetectable, or much lower, than responses against earlier autologous virus [12], [14], [15], [21], [22]. This is especially evident in the initial phase of HIV infection, but the picture may be more complex in chronic infection [23], [24], [25]. It is unlikely that HIV can escape Nabs indefinitely [23].

Here we followed the dynamics of alternating viral neutralization phenotypes over time in a cohort of Men-who-have-Sex-with-Men (MSM) that were monitored from seroconversion up to 1–5 years later. The patients had high set-point viral loads (≥10, 000 RNA copies/ml) and low levels of heterologous Nabs. Confirming previous studies, we see the development of neutralization resistance, including escape from the autologous antibody response. However, we also see the temporal emergence of viruses exquisitely sensitive to both autologous and heterologous Nabs. The heightened neutralization sensitivity was mediated by complex context dependent determinants which include a single arginine residue at position 328 in the stem of V3 in one patient, or a V4 amino acid substitution (T399I, eliminating a putative N-linked glycosylation site) in another patient. These findings extend similar recent observations by Mahalanabis *et al*. [24] and Bosch *et al.* [25] who describe the emergence of neutralization sensitive viruses in patients with broadly neutralizing antibodies or low levels of viremia, indicating that Nabs are unable to fully contain virus replication during chronic infection. This conclusion is further supported by our observation in a dually infected individual, were one of the HIV strains only expanded to detectable levels in blood peripheral blood mononuclear cells (PBMC) after Nabs had developed. The late emerging strain was susceptible to neutralization by antibodies present in the patient's serum prior to its expansion. We propose that the neutralization sensitive viruses are likely to have evolved in an *in vivo* compartment protected from the onslaught of Nabs.

## Results

### Emergence of neutralization sensitive viruses in infected individuals

We examined the evolution of HIV-1 viruses with regard to sensitivity to neutralization in a London cohort of HIV-1 clade B infected men. The patients' likely route of exposure was sexual contact with other men and they experienced a period of symptoms characteristic of primary HIV infection (PHI). The patients were monitored at regular intervals from acute, or early, infection and remained without antiretroviral therapy for the duration of the study.

Multiple sequential *envs* were cloned from seven individuals and inserted into a gp120-deleted clone of HIV-1_HXB2_ [26]. To ensure that only contemporary viruses were analysed *envs* were RT-PCR amplified from plasma at chronic time-points, whereas *envs* from acute infection were amplified from PBMC proviral DNA. The early *envs* were cloned from time-points in close proximity to seroconversion, between 6–32 days after onset of PHI symptoms (Table S1). The later *envs* were cloned from time-points following the development of autologous Nab, the timing of which was assessed using the *envs* cloned from acute infection and reported previously (Table 1; [16], [27]). The breadth of the patient's Nab responses at the second and subsequent cloning dates (days 316–1534) were, however, limited (Table 1). Out of the seven patients studied only three (MM2, MM4 and MM28) had detectable levels of Nabs against the heterologous neutralization sensitive strain HIV-1_IIIB_ (IC_90_s 10–20), and none of the patients had developed Nabs that also could neutralize the more neutralization resistant (Tier 2) strain HIV-1_YU-2_. The patients' viral loads, CD4 cell counts and the exact time-points for *env* cloning are detailed in Table S2.

**Table 1:** Neutralizing activity of sequential serum samples.

Patient:	Virus:	IC_90_ of sera from indicated time-points (days following onset of symptoms):^a^						
**MM1**		**28** b	**48**	**84**	**284**	**494**	**833**	
	Early Env c	<10^d^	nt	<10	<10	20	80	
	IIIB	nt^e^	<10	<10	nt	<10	<10	
	YU2	nt	<10	nt	nt	<10	<10	
**MM2**		**32**	**77**	**113**	**155**	**291**	**484**	**690**
	Early Env	<10	<10	<10	10	40	80	80
	IIIB	<10	<10	<10	<10	10	10	10
	YU2	nt	nt	nt	nt	nt	nt	nt
**MM4**		**17**	**108**	**206**	**297**	**493**	**574**	**833**
	Early Env	<10	<10	20	10	20	20	40
	IIIB	nt	nt	nt	<10	<10	<10	20
	YU2	<10	nt	<10	nt	<10	<10	<10
**MM8**		**12**	**49**	**81**	**333**	**608**	**810**	**957**
	Early Env	<10	<10	10	20	20	40	40
	IIIB	<10	<10	<10	<10	<10	<10	<10
	YU2	<10	<10	nt	<10	nt	<10	nt
**MM23**		**15**	**64**	**113**	**204**	**316**	**498**	**1065**
	Early Env	<10	<10	10	80	160	320	80
	IIIB	<10	<10	nt	<10	10	20	20
	YU2	<10	<10	<10	<10	<10	<10	<10
**MM27**		**28**	**39**	**109**	**299**	**466**	**585**	
	Early Env	<10	<10	<10	<10	10	20	
	IIIB	nt	<10	<10	nt	<10	<10	
	YU2	nt	<10	<10	nt	<10	nt	
**MM28**		**6**	**9**	**62**	**93**	**198**	**405**	**503**
	Early Env	nt	<10	<10	<10	<10	10*^f^	10*
	IIIB	nt	<10	<10	<10	<10	10	20
	YU2	nt	<10	<10	<10	nt	<10	nt

^a^Titres are expressed as the reciprocal dilution of serum required to reduce infectivity by ≥90% (IC_90_) compared to pooled HIV *ve-* serum control.

^b^Serum samples collected from the indicated days after onset of symptoms characteristic of PHI. Time-points from which *envs* were cloned are underlined.

^c^The development of autologous Nabs was assessed against the *envs* cloned from acute infection (days 6–32), as previously reported [16], [27].

^d^<10, less than 90% reduction of infection was observed at the highest serum input tested (1∶10 dilution).

^e^nt, not tested.

^f^*, A clear autologous Nab response had developed by day 405, but it only achieved ∼80% reduction of infection at the highest serum input assayed (1∶10 dilution).

**Figure 1:**
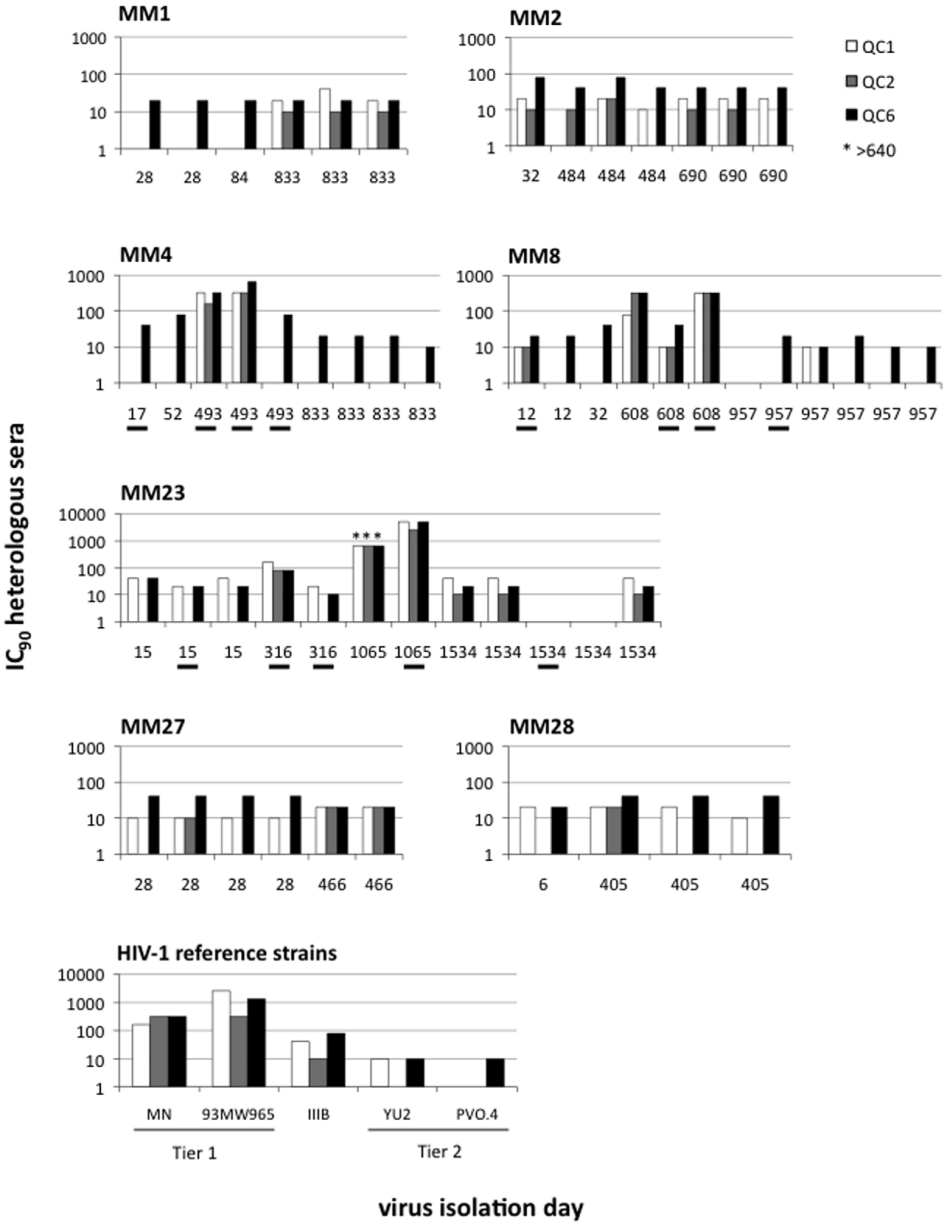
Susceptibility of sequential Envs to neutralization by heterologous sera. The neutralization phenotype of sequential Envs derived from seven HIV-1 infected patients (MM1, 2, 4, 8, 23, 27 and 28), at the indicated days following onset of PHI symptoms, was assessed against three heterologous sera (QC1, 2 and 6) with IC_90_ titres (reciprocal serum end-point dilutions) being reported. Five reference strains were also assayed for comparative purposes. Where less than 90% reduction of infection was observed at the highest serum concentration tested (1:10 dilution) the data is plotted as 1. All patients were infected with neutralization resistant viruses, but highly neutralization sensitive variants (Tier 1 like) emerged during chronic infection in at least three individuals (MM4, 8 and 23). The autologous Nab response to the underlined clones is shown in Fig. 2.

Virus susceptibility to neutralization was assessed using three human sera previously described, QC1, 2 and 6 [16], [26] (Fig. 1). All clones from early infection (days 6–32) were relatively resistant to neutralization with median IC_90_ titres (reciprocal serum end-point dilutions) of 10 against QC1 (range<10–20), <10 against QC2 (range<10–10) and 40 against QC6 (range 10–80). However, within 2 years of infection, viruses from 3/7 patients (MM4, MM8 and MM23) were mixed populations including variants highly sensitive to heterologous serum (IC_90_s between 80–5,120; see day 493 MM4, day 608 MM8 and day 316 MM23). This heightened serum sensitivity was striking even in comparison with the Tier 1 reference strains HIV-1_MN_ and HIV-1_93MW962.25._ IC_90_ titres of the reference sera against HIV-1_MN_ and HIV-1_93MW962.25_ were between 160–320 and 320–2,560, respectively. The Tier 2 clade B reference strains HIV-1_YU2_ and HIV-1_PVO.4_ scored between<10–10. The emergence of neutralization sensitive viruses in the blood was transient in all patients. In patient MM23, however, the neutralization sensitive viruses persisted for an extended period (days 316–1065), becoming exceptionally serum sensitive (IC_90_s>2,560) before ‘reverting’ to a resistance by day 1534. All viruses tested from day 1534 were neutralization resistant, including multiple clones with IC_90_s of<10 against all three sera. A more transient pattern of development of neutralization sensitive variants followed by resistance was seen in MM4 and MM8. All Envs cloned from MM4 and MM8 approximately 1 year after the appearance of sensitive viruses (days 833 and 957, respectively) were neutralization resistant. During the same timeframe, the virus populations in MM1, MM2, MM27 and MM28 appears to have remained neutralization resistant; it is, however, possible that neutralization sensitive viruses arose and subsequently were contained in between the sampling points tested. Regardless, the above experiments demonstrate that neutralization sensitive viruses commonly arise *in vivo*.

**Figure 2:**
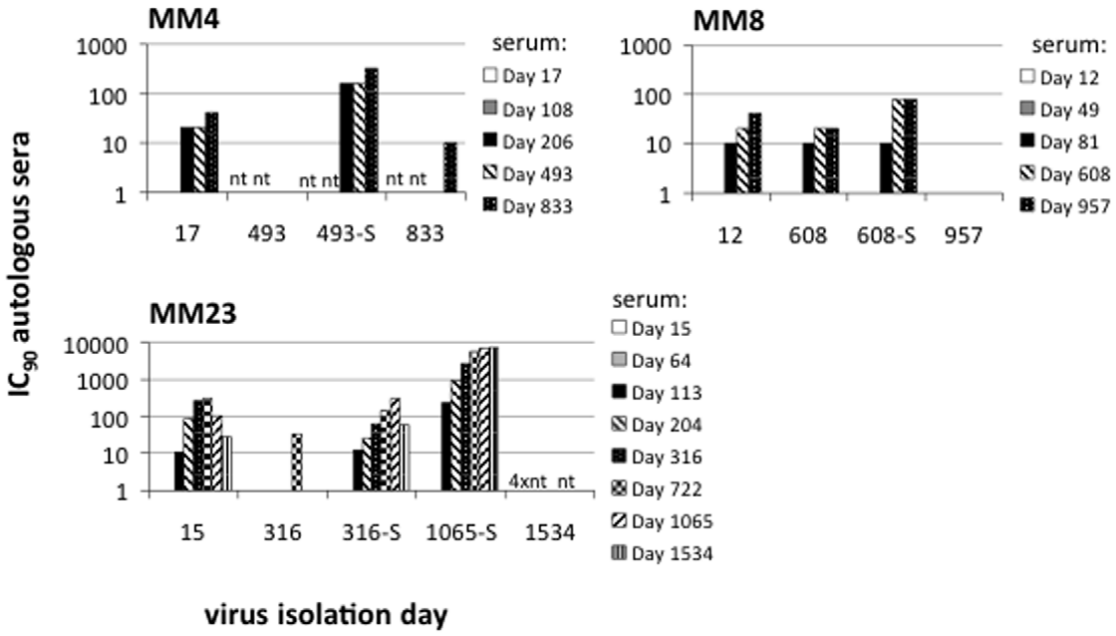
Susceptibility of patient Envs to neutralization by sequential autologous sera. All Envs cloned from patients MM4, MM8 and MM23 (i.e. the patients in which we identified neutralization sensitive virus) were assayed against sequential autologous sera. The IC_90_ titres (reciprocal serum end-point dilutions) are reported for one representative clone from each time-point (underlined in Fig. 1) and viruses that are highly susceptible to neutralization (by heterologous sera) are labelled accordingly (i.e. 493-S, 608-S, 316-S and 1065-S). Due to lack of serum some clones could not be tested against all serum samples. These are labelled accordingly (nt, not tested) and include: MM4 day 493 and 833 viruses which were not tested against day 17 and 108 sera, and MM23 day 1534 viruses which only were assayed against sera from day 316 and 1534. Where less than 90% reduction of infection was observed at the highest serum concentration tested (1∶10 dilution) the data is plotted as 1.

### Neutralization sensitive clones emerge despite being highly susceptible to neutralization by contemporaneous autologous sera

All *envs* cloned from the three patients (MM4, MM8 and MM23) where we identified neutralization sensitive virus were assayed against sequential autologous sera. Data for one or two representative clone from each time-point is shown in Fig. 2. MM4 had developed Nabs by day 206 (IC_90_ 20), MM8 by day 81 (IC_90_ 10) and MM23 by day 113 (IC_90_ 10) (Table 1). As expected, the titre of Nabs against the early (neutralization resistant) Envs increased for a period of several months after their circulation (see day 17 MM4, day 12 MM8 and day 15 MM23; Fig. 2). We also observed the expected pattern of sequential escape from the autologous Nab response by Envs cloned from chronic infection. However, this was not an absolute rule; we found that high susceptibility to neutralization by heterologous serum was reflected in sensitivity to autologous serum and lack of escape. Lack of escape was also noted for one of the neutralization resistant Envs cloned from chronic infection.

The neutralization sensitive Envs amplified from MM4 on day 493 were sensitive to earlier serum from day 206 (IC_90_ 160), to contemporaneous serum (serum taken from the same time-point as the virus, IC_90_ 160) and also later serum (day 833, IC_90_ 320). This was in sharp contrast to the neutralization resistant clone from day 493, which had escaped the autologous Nab response (sera from days 206 and 493 IC_90_<10) and also was resistant to serum from day 833 (IC_90_<10). MM23′s neutralization sensitive viruses were even more striking being neutralized by contemporaneous sera with IC_90_s of 80 (day 316 virus) and >6,000 (day 1065 viruses). MM23′s sensitive viruses were also neutralized by serum from earlier time-points, to either a similar (day 316) or higher (day 1065) degree than the virus cloned from acute infection (day 15). As in MM4 this was in sharp contrast to the co-circulating neutralization resistant clone (from day 316), which had escaped the autologous Nabs in earlier and contemporary serum samples (IC_90_s<10), and only was neutralized by sera from a later time-point (day 722, IC_90_ 30). MM8′s sensitive clones were neutralized by contemporaneous serum (IC_90_ 80), but only slightly more potently than the contemporary (day 608) neutralization resistant clone, which was unusual in that it did not display escape from the autologous Nab response (IC_90_ 20, compared to IC_90_s of 20–80 for Envs from days 12–32). The later MM8 clones, from day 957, were, however, resistant to neutralization by both earlier and contemporaneous sera (IC_90_s<10), indicating that the patient's Nab response was sufficiently potent to impose viral escape by this point. All the later viruses from MM4 (day 833) and MM23 (day 1534) were largely resistant to autologous contemporary sera (IC_90_s≤10), as would be expected from the current model of continuous Nab escape in HIV-1 infection [14], [15], [21], [22].

In summary, we found that all patients initially harboured neutralization resistant viruses which diversified with time and frequently (but transiently) became exceptionally sensitive to neutralization by both autologous and heterologous sera. This occurred despite the presence of Nabs in the blood which were of sufficient potency to drive viral escape. In two patients (MM4 and MM23), Nab escape variants co-circulated with the highly neutralization sensitive viruses. In the third patient (MM8) Nab escape was only observed at a later sampling point, but it is possible that concurrent escape was missed due to the limited sampling of resistant Envs from the time-point when the neutralization sensitive variants emerged (one clone was tested). Thus, neutralization sensitive Envs arise despite onslaught from Nabs in the blood.

### Phylogenetical analysis of envelopes – identification of a dual infection

**Figure 3:**
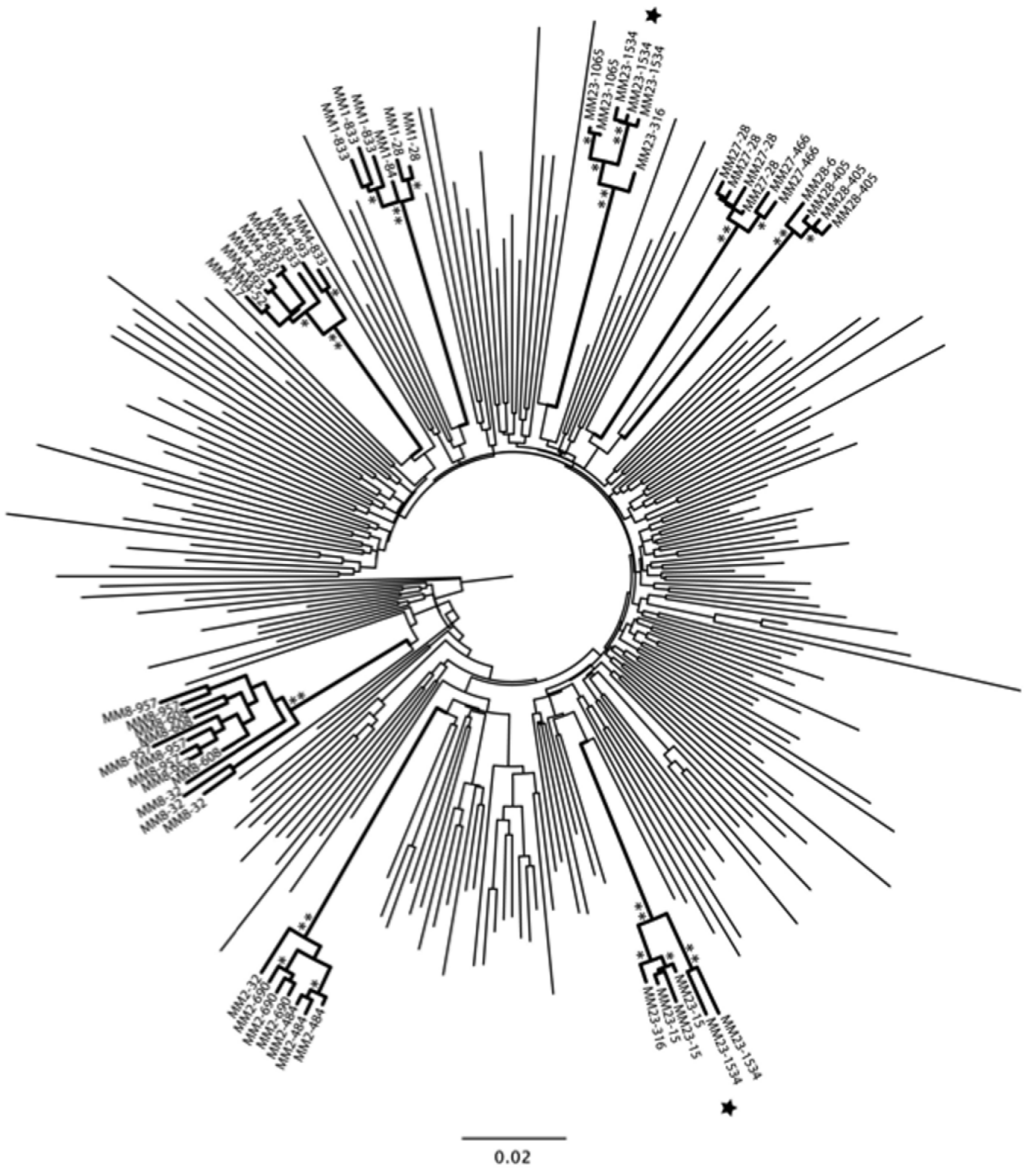
Phylogenetic relationship between cloned *envs.* Maximum-likelihood tree representing the phylogenetic relationship between 56 HIV-1 partial (1937 bp) *env* gene sequences from seven HIV-infected patients from the UK and 200 HIV-1 subtype B *env* gene sequences extracted from the LANL HIV database. The tree was reconstructed according to the GTR+I+G model of nucleotide substitution. Branch lengths are expressed as the number of nucleotide substitution per sites, with branches leading to the clones generated herein indicated in bold. Percent bootstrap support values above 90%, or of 100%, are indicated by one or two asterisks, respectively, on the corresponding branches. The *envs* are labelled with patient ID followed by isolation day (e.g. MM1-28, *env* from MM1 day 28). Clones from patient MM23 separates into two distinct clusters (marked with stars), indicating that the patient was infected with two different clade B strains.

The *env* genes cloned from the seven patients were sequenced and a maximum-likelihood a tree was generated (Fig. 3). For robustness 200 additional clade B *envs* from the Los Alamos National Laboratory (LANL) database were included. *Envs* from 6/7 patients, including MM4 and MM8, formed single clusters with bootstrap values of 100% for each patient confirming the phylogenetical relationship between the clones. However, the clones from patient MM23 formed two separate clusters (indicated with stars, Fig. 3), and their amino acid sequences differed by approximately 25% (not shown). This indicates that MM23 was infected with two distinct stains; strain A which was cloned from days 15, 316 and 1534 and remained neutralization resistant, and strain B that only amplified from day 316 onwards, and was highly sensitive to neutralization when first detected (at day 316 and 1065).

### Characterisation of the dual infection of MM23

The dual infection of patient MM23 afforded us the opportunity to follow the evolution of two distinct viral quasispecies. In the above experiments strain B was only amplified from plasma samples taken a year, or more, after the appearance of strain A. We first sought to define whether MM23 was superinfected (i.e. if infection first occurred with strain A and later with stain B) or if both strains infected MM23 prior to seroconversion.

**Figure 4:**
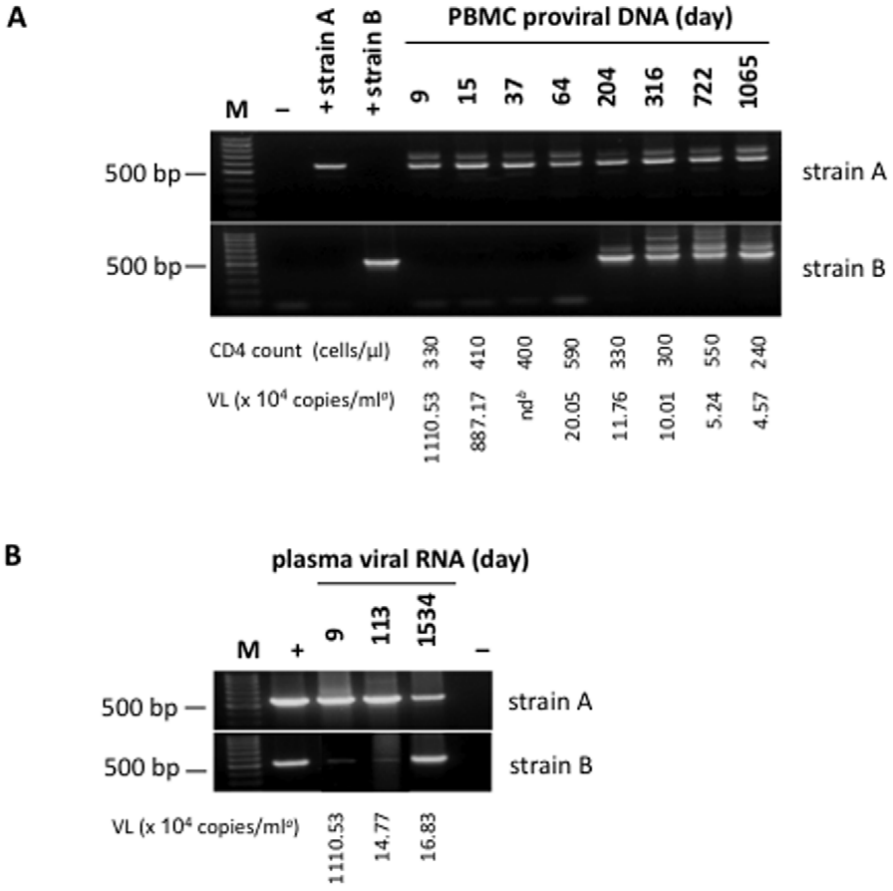
MM23 strain specific nested PCR. Strain specific primers for nested PCR were designed to amplify ∼500 bp segments of the *env* gene of MM23′s viruses. The PCR conditions were optimised on plasmids encoding previously cloned *envs* and then applied to PBMC proviral DNA (Fig. A), or plasma viral RNA (Fig. B), from indicated days after onset of PHI.symptoms. (**A**) Strain A is detectable in PBMC from all time-points assayed (expected size of PCR product 577 bp), whereas strain B is only detectable from day 204 onwards (expected size of PCR product 524 bp). (**B**) Strain A is detectable in plasma samples from all time-points assayed, and likewise is strain B. The marker (M) is the BenchTop 100 bp DNA ladder (Promega, UK), which contains a 500 bp band of triple intensity. Positive controls (+) were plasmid pHXB2*env*23.2.E (for strain A) and pHXB2*env*23.8.12 (for strain B). As a negative control (–) proviral DNA or viral RNA was replaced with dH_2_O. Footnotes: *^a^* Plasma viral load (VL) determined using Chrion 3.0 (Emeryville, Cal., USA); *^b^* nd, not determined.

Primers were designed for nested PCR to differentially amplify either strain A or B, and applied to proviral DNA extracted from PBMC and plasma viral RNA (Fig. 4). Strain A was detected at all time-points, from day 9 to 1534, both in PBMC and plasma samples (Fig. 4A and B). Strain B was also detected in plasma samples from day 9 onwards (Fig. 4B). Hence, MM23 was most likely infected with both strains around the same time-point, which is consistent with the case history of the patient.

Interestingly, even though the PCR could efficiently and specifically amplify strain B, we could not detect it in PBMC until day 204 (Fig. 4A). Comparison of the PCR products in Fig. 4 indicates that strain B was a ‘minor species’ compared to strain A, at least in the blood, before day 204. Thus, although MM23 was dually infected, only strain A established itself (at a detectable level) in PBMC in the initial phase of the infection. The outgrowth of strain B (occurring between days 113 and 204) was not associated with a fall in CD4 cell numbers or an increase in viral load, although we can not exclude that a transient change (’blip’) may have occurred in between the sampling points (Fig. 4). Likewise, no fluctuation in viral load or CD4 numbers was seen upon the emergence of neutralization sensitive viruses in patients MM4 and MM8 (Table S2).

### Expansion of strain B occurred following induction of a specific Nab response

Having established that strain B was present in patient MM23 from seroconversion, we questioned the role of Nabs in the dynamics of its evolution. The data on the neutralizing activity of chronological autologous serum samples was re-analyzed with attention on the strain identity of the different Env clones (Table 2A). To get a better understanding of the breadth of the Nab response, MM23′s sera were also assayed against five heterologous HIV strains with varied susceptibility to neutralization (Table 2A). Serum Nabs were first detectable at day 64, against the highly neutralization sensitive Tier 1 virus HIV-1_93MW965.25_ (IC_90_ 11), but these early Nabs were unable to neutralize any of the other heterologous strains or any of the MM23 viruses (IC_90_s<10). Day 113 serum neutralized autologous strains A and B; 2/3 virus A clones derived from day 15 (IC_90_ 11-12), and virus B from days 316 (IC_90_ 12) and 1065 (IC_90_>40). By day 204, when strain B was first detected in PBMC, the potency of the Nab response had increased slightly so that the IC_90_ for the earliest strain B clone assayed was 26 (clone 23.8.12 from day 316). The serum from day 204 displayed no neutralizing activity against the equally neutralization sensitive strains HIV-1_MN_ and HIV-1_IIIB_ (or against the more neutralization resistant strains HIV-1_YU2_ and HIV-1_PVO.4_; for comparison of viruses susceptibility to neutralization see Fig. 1). Hence, a Nab response specific to strain B had evidently developed prior to the strains expansion into (or in) PMBC but this response was unable to contain the virus replication *in vivo*. Indeed, although contemporaneous serum samples failed to neutralize stain A throughout the study, as has been reported as a characteristic of HIV infection [14], [15], [21], [22], the picture was different for strain B. Virus from day 316 (23.8.12) was inhibited by serum from the same time-point (IC_90_ 64), and viruses from day 1065 (23.12.3/7) were extremely sensitive to contemporary serum (IC_90_>6,000). Escape from the autologous Nab response by strain B was detectable at day 1534, when viruses (23.13.1/4/16) were no longer neutralized by serum from earlier time-points (day 316, lack of serum precluded additional sampling), and only weakly neutralized by contemporary sera (IC_90_s 11–17). This escape from autologous neutralization coincided with loss of sensitivity to heterologous serum (IC_90_s 10–40, Fig 1).

**Table 2:** Susceptibility of MM23′s viruses to neutralization by autologous sera, sCD4 and MAbs.^a^

		A IC_90_ (reciprocal serum dilution)									B IC_50_ (µg/ml)				
		Autologous serum from days:^b^													
Virus:		15	37	64	113	204	316	722	1065	1534	sCD4	IgG1b12	17b(+sCD4)^f^	17b(−sCD4)	2G12
*Autologous virus:*	*Clone* c *:*														
Day 15 strain A	23.2.D	<10^d^	<10	<10	<10	51	254	215	77	nt^e^	>15^g^	>15	N/A^h^	>15	>15
	23.2.E	<10	<10	<10	11	87	263	306	101	27	>15	>15	N/A	>15	>15
	23.2.H	<10	<10	<10	12	84	148	154	81	nt	>15	>15	N/A	>15	>15
Day 316 strain A	23.8.18	<10	<10	<10	<10	<10	<10	33	<10	<10	>15	>15	N/A	>15	5.1
strain B	23.8.12*	<10	<10	<10	12	26	64	139	303	59	1.1	0.6	7.2	6.8	3.7
Day 1065 strain B	23.12.3*	<10	<10	<10	>40	39	100	5,352	7,742	6,238	0.06	0.07	0.2	0.3	>15
	23.12.7*	<10	<10	<10	235	907	2,627	5,618	6,992	7,240	0.05	0.07	0.2	0.6	>15
Day 1534 strain A	23.13.5	nt	nt	nt	nt	nt	<10	nt	nt	<10	>15	>15	N/A	>15	>15
	23.13.14	nt	nt	nt	nt	nt	<10	nt	nt	<10	>15	>15	N/A	>15	>15
strain B	23.13.1	nt	nt	nt	nt	nt	<10	nt	nt	14	0.4	0.08	0.7	1.0	>15
	23.13.4	nt	nt	nt	nt	nt	<10	nt	nt	11	nt	nt	nt	nt	nt
	23.13.16	nt	nt	nt	nt	nt	<10	nt	nt	17	1.7	0.2	0.6	2.8	>15
*Heterologous virus:*															
MN	Tier 1	<10	<10	<10	<10	<10	13	nt	23	13	0.2	0.2	0.01	>15	>15
93MW962.25	Tier 1	<10	<10	11	47	332	nt	nt	3,241	2,935	nt	nt	nt	nt	nt
IIIB		<10	nt	<10	nt	<10	10	nt	20	21	nt	nt	nt	nt	nt
YU-2	Tier 2	<10	nt	<10	nt	<10	<10	nt	<10	<10	nt	nt	nt	nt	nt
PVO.4	Tier 2	nt	nt	<10	nt	nt	nt	nt	nt	<10	12.1	>15	>15	>15	5.6

^a^Titers are expressed as: (**A**) the reciprocal dilution of serum required to reduce infectivity by ≥90% (IC_90_) compared to pooled HIV *ve-* serum control; or (**B**) the concentration of sCD4 or MAb required to reduce infectivity by ≥50% (IC_50_) compared to medium only control.

^b^MM23 serum samples collected from the indicated days after onset of symptoms characteristic of PHI.

^c^Clones indicated with an asterisk (*) displayed high sensitivity to neutralizzation by heterologous sera.

^d^<10, less than 90% reduction of infection was observed at the highest serum input tested (1:10).

^e^nt, not tested.

^f^Viruses were assayed for neutralization by MAb 17b both in the presence (+sCD4) and absence (−sCD4) of sCD4. When sCD4 was included with 17b reduction in infection was calculated against virus assayed in the presence of sCD4 alone. The amount of sCD4 included varied between viruses, being equal to the IC_50_ for each virus.

^g^>15, less than 50% reduction of infection was observed at the highest (15 µg/ml) concentration tested.

^h^N/A, not assessed as the virus was resistant to neutralization by sCD4.

### Neutralization sensitivity is associated with exposure of the CD binding site and CD4-induced epitopes

In addition to assessing the MM23 viruses' susceptibility to neutralization by sera we also tested them against a panel of NMAbs (IgG1b12, 2G12 and 17b) and soluble CD4 (sCD4; Table 2B). Strain A was resistant to all reagents except 2G12 (IC_50_s>15 µg/ml). In contrast, the serum sensitive clone of strain B from day 316 (23.8.12) was neutralized by sCD4, IgG1b12 and 2G12 with IC_50_ values<5 µg/ml; and, interestingly, with a similar potency by 17b in the absence (IC_50_ 6.8 µg/ml) or presence (IC_50_ 7.2 µg/ml) of sCD4. The exceptional serum-sensitivity of clones from day 1065 (23.12.3/7) was reflected in their susceptibility to neutralization by sCD4 (IC_50_ 0.05–0.06 µg/ml), IgG1b12 (IC_50_ 0.07 µg/ml) and 17b (presence and absence of sCD4; IC_50_s 0.2–0.6 µg/ml). Envs 23.12.3/7 were, however, resistant to neutralization by 2G12, presumable due to an amino acid substitution [aspagarine (N) to serine (S)] at the 2G12-associated N-linked glycosylation site (NLGS) at residue N332 (data not shown) [28]. The IC_50_ values for strain B 23.12.3/7 were within the same range as those for the Tier 1 strain HIV-1_MN_, except that 17b only neutralized HIV-1_MN_ in the presence of sCD4. However, somewhat surprisingly, the late (day 1534) serum resistant strain B clones (23.13.1/16) displayed similar susceptibility to neutralization by sCD4, IgG1b12 and 17b (IC_50_s 0.08–2.8 µg/ml) as the earlier sensitive Envs. The serum sensitive viruses from patients MM4 and MM8 were, like those of MM23, neutralized by 17b in the absence of sCD4 (IC_50_s 4.0–8.0 µg/ml), but more potently with sCD4 (IC_50_s 0.01–0.03 µg/ml; not shown). They were also highly sensitive to neutralization by either sCD4 (MM4, IC_50_s 0.2 µg/ml) or IgG1b12 (MM8, IC_50_s 0.2–0.3 µg/ml; not shown).

### Neutralization sensitive phenotype confirmed by SGA

As the *envs* were cloned by conventional PCR, the neutralisation sensitive phenotype observed in chronic infection may be due to PCR errors such as recombination between different genomes during amplification. Additionally, although most Nabs target the surface unit of Env (gp120), the interaction of the patients' gp120s with a heterologous transmembrane unit (HIV-1_HXB2_ gp41) in the vector system we used to generate virus could possibly affect their phenotype. To address these concerns we re-amplified full-length gp160 sequences from MM4 day 493 and MM8 day 608 by single-genome-amplification (SGA) [29]. Virus was then produced by pseudotyping an *env*-deleted HIV genome with the gp160 clones.

Sequence data was obtained for 34 SGA-derived *envs* from MM4 and 21 from MM8. All but two of the clones were unique and none of them were identical to the original *envs* cloned into the HXB2-vector (Figure S1).

**Figure 5:**
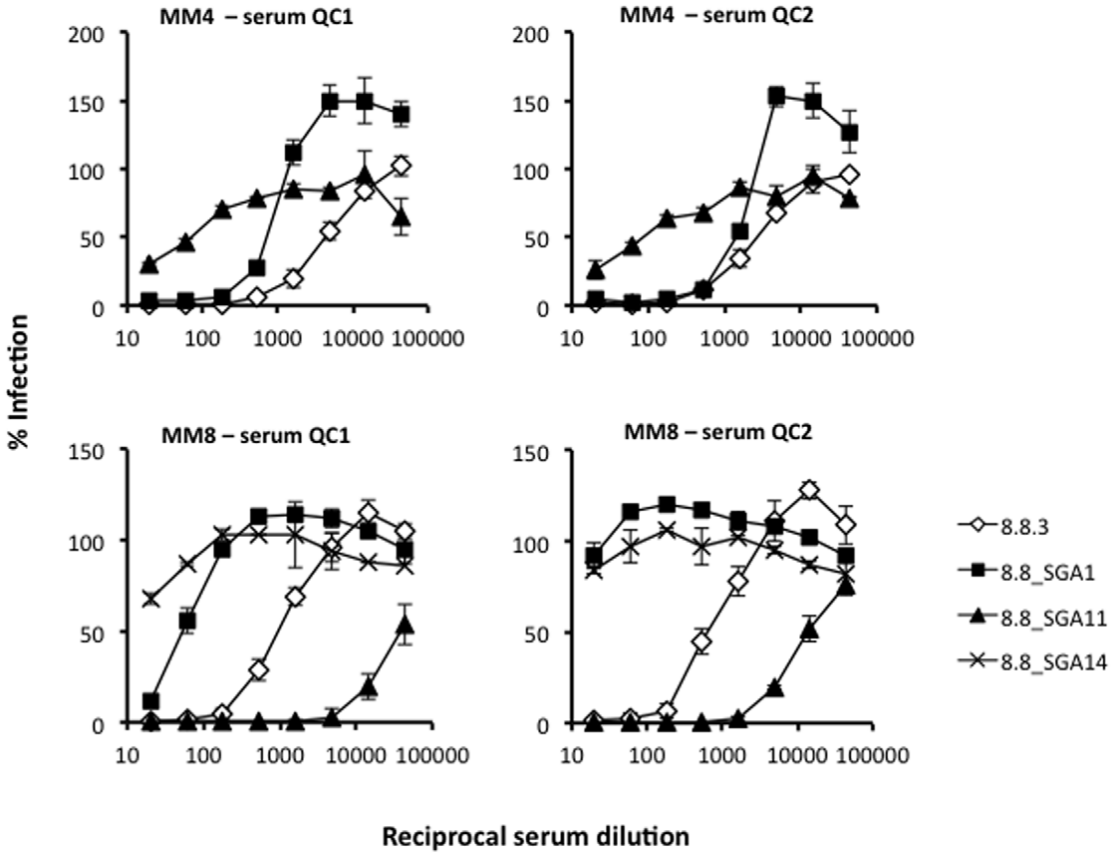
Neutralization phenotype of SGA derived gp160 Envs. The neutralization phenotype of SGA derived full-length Envs was assessed against heterologous sera QC1 and 2 alongside neutralization sensitive HXB2-gp120 chimeras. Top panels display data for Envs from MM4 day 493; SGA clone 4.10_SGA9 was almost as neutralization sensitive as the original HXB2-gp120 chimera 4.10.3 whereas clone 4.10_SGA29 displayed a neutralization resistant phenotype (IC_90_s<20). Lower panels display data for Envs from MM8 day 608; one out of three SGA clones tested (8.8_SGA11) was hyper sensitive to neutralization. The graphs display data from one representative titration, with error bars representing the standard deviations between replicates.

Two SGA-cloned Envs from MM4 and three from MM8 were selected for assessment in neutralisation assays and tested against heterologous sera (QC1 and 2) alongside the original neutralization sensitive gp120-chimeras 4.10.3 (MM4) and 8.8.3 (MM8) (Fig. 5). One of the two SGA-cloned Env-pseudotypes from MM4 (4.10_SGA9) was neutralization sensitive (IC_90_s 180), with titres comparable to that of the previously characterised chimera derived from the same time-point (4.10.3, IC_90_s 540). The other clone was neutralization resistant (4.10_SGA29, IC_90_s<20). One of three SGA-derived Env-pseudotypes from MM8 was also highly neutralisation sensitive (IC_90_s 1,620-4,860), while the other clones were neutralization resistant (8.8_SGA1/14, IC_90_s<20). This data confirms our original observation of a mixed population of neutralization sensitive and neutralization resistant viruses in chronic infection in MM4 and MM8.

### Complex determinants of neutralization sensitivity

**Figure 6:**
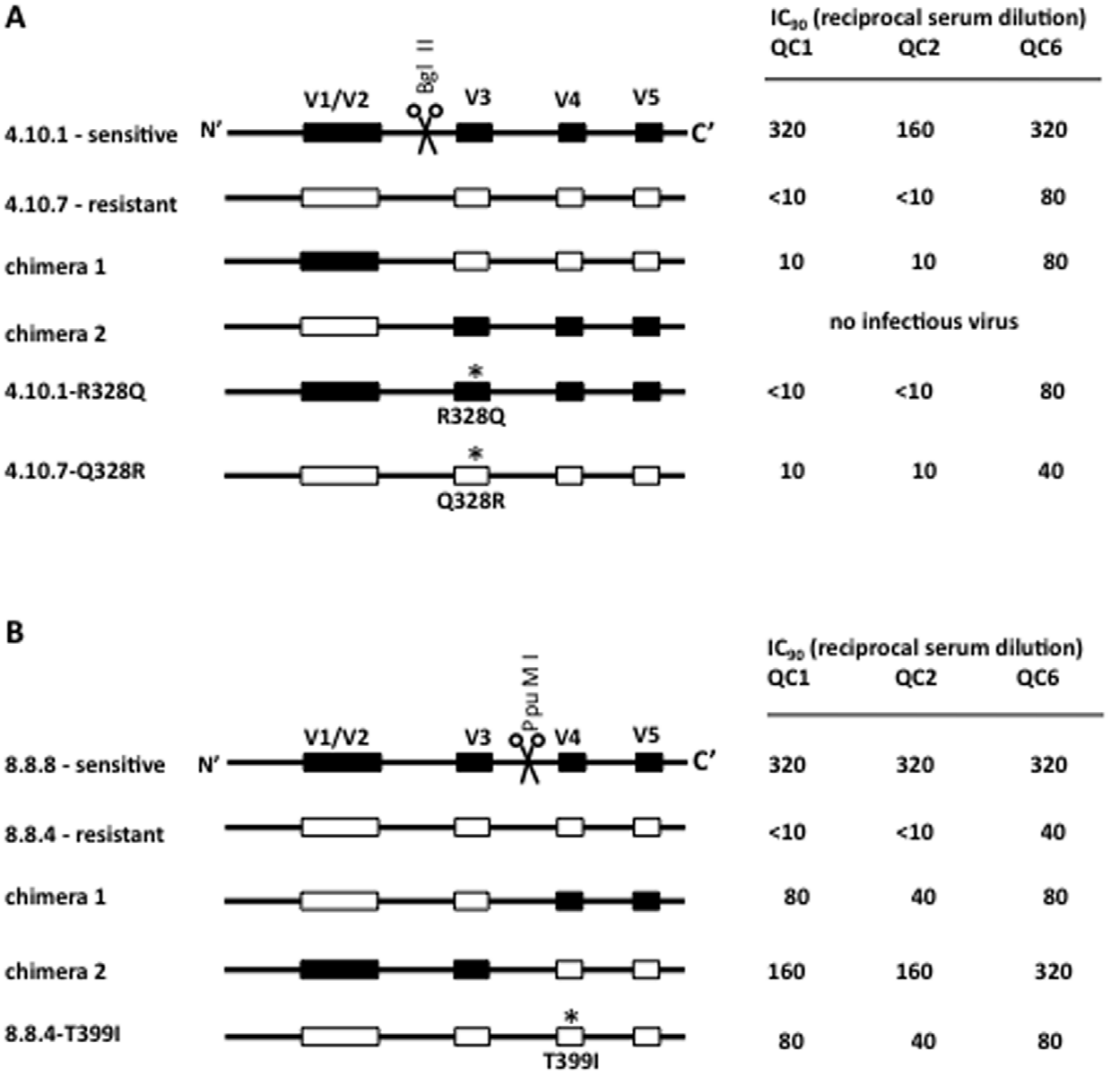
Molecular determinants of heightened neutralization sensitivity. To map molecular determinants of the neutralization sensitive phenotype sections of the *env* gene was swapped between neutralization sensitive (black boxes) and neutralization resistant (white boxes) gp120 clones from MM4 day 493 (A) and MM4 day 608 (B). This was done by exploiting conserved Bgl II or PpuM I restriction enzyme sites (indicated in the figure) in combination with restriction sites incorporated in the primers used for gp120 cloning (see Figure S1 for details). Further mapping was done by SDM, altering amino acid residues indicated with asterisks. The phenotype of the chimeras and the mutants was assessed against heterologous sera (QC1, 2 and 6) with IC_90_ titres (reciprocal serum end-point dilutions) being reported. (A) The neutralization sensitive phenotype of MM4 clone 4.10.1 was dependent on an unusual arginine (R) residue in the stem of V3 (4.10.1-R328Q). However, introduction of this residue was not sufficient to infer sensitivity on the resistant MM4 clone 4.10.7 (4.10.7-Q328R). (B) In MM8, neutralization sensitivity determinants were present on both sides of the PpuM I site (chimera 1 and 2) and included a threonine to isoleucine change at residue 399 in V4 (compare 8.8.4 and 8.8.4-T399I).

Comparison of amino acid sequences changes between neutralization resistant and sensitive Env clones did not reveal any obvious amino acid residues common to all sensitive Envs. Therefore, we attempted to identify molecular determinants of neutralization sensitivity by swapping Env regions followed by site-directed mutagenesis (SDM), using Envs from patients MM4 and MM8. Fig. 6 shows a schematic of the Env swaps and SDM used to map determinants of neutralization sensitivity and resistance.

Introduction of the ‘V3-V5’-region (residues 274–503) from the neutralization resistant MM4 clone (4.10.7) into the sensitive MM4 clone (4.10.1) resulted in neutralization resistance (chimera 1, Fig. 6A). The reverse swap was lethal (chimera 2, Fig 6A). This demonstrates that the ‘V3–V5’-region contains key determinants of sensitivity/resistance to serum neutralization. We next determined the contribution of an amino acid substitution in the stem of V3 (residue 328). Residue 328 is a basic amino acid (arginine, R, or lysine, K) in the sensitive MM4 Envs but a polar amino acid (glutamine, Q) in most other clones, in agreement with the consensus for clade B viruses (Figure S1A). The R328Q change in the sensitive Env conferred serum resistance (4.10.1-R328Q). However, reverse mutagenesis in the resistant Env had no effect (4.10.7-Q328R). This indicates that the V3 loop is the major determinate of neutralization sensitivity/resistance in MM4, but also that additional residues are important.

In MM8 the introduction the ‘V4–V5’-region (residues 369–503) of the sensitive Env (8.8.8) into the resistant clone (8.8.4) conferred partial neutralization sensitivity (chimera 1, Fig 6B). The reverse swap, however, had little or no effect on the neutralization phenotype (chimera 2), indicating the presence of sensitising amino acid residues in both halves of Env. We next focused on a threonine (T) to isoleucine (I) mutation at residue 399, which eliminates a putative NLGS in V4, and is conserved between all neutralization sensitive MM8 Envs (Figure S1B). This residue has been previously implicated in neutralization escape [14]. The T399I mutagenesis in the resistant MM8 Env conferred partial sensitivity, replicating the phenotype of chimera 1 (8.8.4-T399I, Fig. 6B). Regrettably we were unable to pinpoint neutralization sensitising determinants in the N-terminal half of MM8′s Env (data not shown). Hence, in our patient sets we found that determinants of neutralization sensitivity are complex but that residues within V3 and V4 are important.

### Viral tropism - lack of association with macrophage infectivity

Others have reported emergence of neutralization sensitive viruses during transition in coreceptor usage from CCR5 (R5) to CXCR4 (X4) [30]. In our study clinical progression and coreceptor switch was only observed in MM8, where a mixed population of R5-only and R3/R5/X4-tropic Envs were cloned a year after (on day 957) the appearance of neutralization sensitive viruses [31]. The earlier viruses from MM8, including the neutralization sensitive clones, and also all Envs from MM4 and MM23, were R5/R3-tropic (Table 3, [31], [32]). Another possible explanation for the emergence and persistence of MM23 strain B despite its high sensitivity to neutralization by autologous (and heterologous) sera could be that the strain replicated in a Nab protected niche. We tested the ability of a subset of MM23′s viruses to infect primary Monocyte-Derived-Macrophages (MDM). Only strain B-23.13.4, a day 1534 neutralization resistant clone, was able to infect these cells (Table 3). Likewise, there was no association between tropism for MDM and sensitivity to neutralization in patients MM4 and MM8 (Table 3). Indeed, the two (day 493) neutralization sensitive Envs cloned from MM4 displayed marked differences in macrophage tropism, as did the two (day 608) neutralization sensitive Envs cloned from MM8 (Table 3). The neutralization resistant MM4 and MM8 viruses ranged from being highly macrophage tropic (achieving similar titres on NP-2/CD4/CCR5 cells and MDM) to being non-MDM tropic (no or minimal levels of MDM infection). These observations indicate that the development of neutralization sensitive viruses occurs in the absence of clinical progression or coreceptor switch, and without any link to macrophage tropism.

**Table 3:** Coreceptor usage and tropism for MDM.

Patient	Env isolation day^a^	Env clone	Neutralization phenotype^b^	Coreceptor usage^c^	MDMtropism^d^
MM4^e^	17	4.1.33	R	R5/R3	−
	493	4.10.1	S	R5/R3	−
	493	4.10.3	S	R5/R3	++
	493	4.10.7	R	R5/R3	++
	844	4.12.1	R	R5/R3	(+)
MM8^e^	12	8.2.50	R	R5/R3	+++
	12	8.2.51	R	R5/R3	+
	608	8.8.3	S	R5/R3	++
	608	8.8.4	R	R5/R3	+++
	608	8.8.8	S	R5/(R3)	(+)
	608	8.9.D	R	X4/R5/R3	++
	608	8.9.I	R	R5	+
MM23	15	strain A- 23.2.E	R	R5/R3	−
	316	strain A- 23.8.18	R	R5/R3	−
	1534	strain A- 23.13.14	R	R5/(R3)	−
	316	strain B- 23.8.12	S	R5/R3	−
	1065	strain B- 23.12.7	S	R5/(R3)	−
	1534	strain B -23.13.4	R	R5/(R3)	+

^a^Time-point from which the patient *env* was amplified, in days from onset of symptoms characteristic of PHI.

^b^Virus susceptibility to neutralization by heterologous sera (see Fig. 1); S, sensitive: R, resistant.

^c^Coreceptor tropism assessed on NP-2/U87 indicator cells expressing CD4 and one of the putative coreceptors CCR5, CXCR4 and CCR3. Coreceptors supporting viral entry within 1 log_10_ (or in brackets 2 log_10_) of the level seen with the preferred coreceptor are listed; R5, CCR5; R3, CCR3; X4, CXR4.

^d^Assessed by p24 immunostaining four days post-inoculation. The tropism for MDM is scored relative to the virus titre on NP-2/CD4/CCR5 cells; +++ titre on MDM>90% of the titre on NP-2 cells; ++ >10%: + >1%; (+) >0.1%; -, no detectable infection or <0.1% of titre on NP-2 cells.

^e^The coreceptor usage of the Envs from acute infection and the first chronic infection time-point has been reported previously [31], [32], as have the data on MDM tropism for MM4′s and MM8′s viruses [50].

## Discussion

Here we show that despite only neutralization resistant HIV-1 viruses being detectable in primary infection, viruses highly sensitive to neutralization arise over time *in vivo*. The outgrowth of such neutralization sensitive viruses occurs even though potent Nabs are present in serum.

Our results, at first, are apparently at odds with previous studies demonstrating the development of neutralization escape rather than sensitivity *in vivo* [14], [15], [22]. But to the contrary, we confirm and extend these observations. We confirm the ability of contemporaneous virus to escape autologous neutralization and further show the development of neutralization sensitive viruses, sometimes concordantly with neutralization escape. In some cases neutralization sensitive viruses persist in the blood for many months before resistance develops.

Our observation of emergence of neutralization sensitive viruses in a cohort of clade B infected MSM complements other recent studies which have reported similar emergence of neutralization sensitive variants during chronic infection in (1) clade B infected (elite) *viremic controllers* with moderately broad Nab responses [24] and (2) in one clade A infected individual with an exceptionally *broad Nab* response [25]. In both our study and in those of Mahalanabis *et al*. [24] and Bosch *et al*. [25], neutralization sensitive variants emerged despite being susceptible to neutralization by contemporaneous sera and sera from (several months) earlier in infection. We show that the hypersensitive phenotype is unlikely to be an *in vitro* artefact by employing SGA and cloning of full-length *envs*. Together these reports provide evidence that although Nabs shape the viral quasispecies they do not invariably drive escape in chronic HIV infection. Neutralization sensitive viruses emerge in patients with a wide range of viral loads and a broad spectrum of Nab responses. Furthermore, in the case of the dually infected patient MM23 a specific (strain recognising) Nab response of reasonable potency was unable to prevent the late expansion of strain B into PBMC.

The molecular determinants for the neutralization sensitive phenotype were found to be complex and context dependent. An amino acid substitution in the stem of the V3-loop (Q328R) was identified as essential, but not sufficient, for the sensitive phenotype of MM4′s viruses. In MM8′s virus, the neutralization sensitive phenotype was partly due to a threonine to isoleucine change in V4 (T399I). This amino acid change eliminates a putative NLGS that previously have been implicated in Nab escape [14]. Hence, in our patient set we found that residues within V3 and V4 impacted on virus susceptibility to neutralization.

The observations made in this report raise several questions, including to what extent the neutralization sensitive variants contributes to the contemporary and subsequent composition of the viral quasispecies. The ease by which we identified sensitive clones indicates that (at times) neutralization sensitive variants could account for a sizable fraction of the replicating viral population. Furthermore, the *in vitro* infectivity of the neutralization sensitive viruses was comparable to that of the neutralization resistant variants, both with regard to the titres obtained and the number of infectious units per picogram of reverse transcriptase (data not shown).

A second major question raised by this study is how the neutralization sensitive viruses can emerge. One possibility is that the neutralization sensitive clones co-opt Nabs to promote infection of cells expressing Fc- (or complement-) receptors [34]. We addressed this possibility by titrating virus on primary MDM in presence of serial-diluted autologous sera, but we found no indication of enhanced infection (data not shown). Hence, our data does not support this scenario.

We prefer the hypothesis that the neutralization sensitive viruses evolve in a compartment ‘protected’ from neutralization or an immune-privileged site. Possible immune privileged sites with relevance to HIV infection include the CNS/brain and the gonads (testis) [35]. Growth in the absence of Nabs could favour viruses with better replication kinetics perhaps due to a more ‘open’ CD4-binding region. The scenario is akin to T cell line adaptation of HIV-1 *in vitro*. T-cell line adapted viruses are typically much more sensitive to Nabs, particularly to the CD4-binding region [33]. In support of this notion, all Envs with heightened serum sensitivity were potently neutralized by sCD4 and/or IgG1b12. Neutralization by 17b in the absence of sCD4 was also observed, which could indicate a more exposed co-receptor binding site. In contrast, out of nineteen serum resistant *env*-chimeras tested from this patient group (MM1-MM28) only three were neutralized by 17b in absence of sCD4.

The kinetics of viral replication in patient MM23 also support the hypothesis that sensitive strains may evolve in a separate compartment. Phylogenetic analysis demonstrated that MM23 was infected with two distinct strains. Strain A was detectable in both plasma and PBMC throughout the study and remained neutralization resistant. Strain B, however, was a ‘minor species’ in the plasma in early infection and could only be detected in PBMC after day 204. This suggests that strain B was replicating in a separate compartment during the initial phase of the infection.

It is well known that HIV is able to infect the CNS/brain with macrophage tropism often predicting the ability of primary HIV-1 strains to replicate in microglia [37], [38]. Most likely the virus enters the CNS mainly through infected monocytes and macrophages, or lymphocytes [36], when viral replication is at its highest, at peak primary viremia. Duenas-Decamp *et al.* have shown that epitopes that determine sensitivity to IgG1b12 affect macrophage tropism and the ability to use low levels of CD4 [39]. This lead us to question whether the observed serum sensitivity was associated with macrophage tropism, but we found no link. Thus, if neutralization sensitivity is associated with protected replication in the brain we could find no evidence that this additionally relates directly to macrophage tropism. With further regard to tropism, Bunnik *et al.* have reported an association between neutralization sensitivity and emergence of CXCR4 use [40]. In agreement with this, clinical progression and coreceptor switch was observed in one patient (MM8) after neutralization sensitive virus emerged. However, we observed no evidence of coreceptor switch in the rest of the cohort. A growth advantage to particular tissue subsets of T cells, or macrophages, remains a possibility. It is also possible that neutralization sensitivity has limited effect on replication in an *in vivo* compartment where direct cell-to-cell spread could be a primary mode of transmission.

In summary, with regard to humoral immunity, longitudinal studies reveal a continually evolving virus that circumvents the host Nab response [14], [15], [21], [22]. However, the sensitivity or resistance to neutralization may not only depend on time and the development of Nabs but also be dependent on location. The interplay of selective forces for greater virus replication efficiency resulting in sensitivity to Nabs occurring in a compartment protected from antibody surveillance could explain the temporal course described here for the *in vivo* emergence of HIV-1 variants with potent sensitivity to Nabs. Further studies are needed to evaluate this possibility, and define to what extent neutralization sensitive variants shape the viral quasispecies.

## Materials and Methods

### Patient cohort and Ethics statement

We analysed HIV-1 Envs derived from seven HIV-1 subtype B infected men whom presented at a London clinic with symptomatic primary HIV infection Blood samples were obtained at seroconversion and thereafter at regular intervals up to five years after infection (405–1534 days after onset of symptoms). Recent HIV-1 infection was diagnosed by the detection of HIV-1 genomes (PBMC proviral DNA or plasma RNA) in the presence, or absence, of an evolving antibody profile that subsequently became fully positive (Table S1). All subjects initially declined antiretroviral therapy and remained treatment naïve throughout the study. The study protocol was approved by the Camden and Islington NHS Trust Ethics Committee and written informed consent obtained from all subjects.

### Viruses, sera and MAbs

HIV-1_MN_ and HIV-1_IIIB_ were obtained from the Centralised Facility for AIDS Reagents (CFAR), National Institute for Biological Standards and Controls (NIBSC), UK, and propagated in C8166 and H9 cells, respectively, also obtained from the CFAR. The 93MW965.26 *env* clone was provided by D Montefiori (Duke University Medical Center, USA) through the Comprehensive Antibody Vaccine Immune Monitoring Consortium (CA-VIMC), as part of the Collaboration for AIDS Vaccine Discovery (CAVD). The PVO.4 *env* clone [41] was obtained through the NIH AIDS Research and Reference Reagent Program (USA). Viruses 93MW965.26 and PVO.4 were produced in 293T/17 cells obtained from the ATCC (LGC Standards, UK) by co-transfection with their respective plasmid constructs and the pSG3Δenv plasmid (obtained through the NIH AIDS Research and Reference Reagent Program, [14], [42]). MAb IgG1b12 was kindly provided by D Burton (The Scripps Research Institute, USA), MAb 17b by R Wyatt (Vaccine Research Center, NIH, USA) and recombinant sCD4 by I Jones (The University of Reading, UK). MAb 2G12 was obtained from Polymun Scientific GmbH, Austria. QC sera 1, 2 and 6 from HIV-1-seropositive individuals have been described previously [26]. Both the reference sera and autologous patient sera were heat-inactivated (56°C, 1 hr) before use.

### Amplification of gp120 and generation of infectious molecular clones

Viral envs from acute/early infection had been amplified from proviral DNA and infectious molecular clones generated by inserting the envs into the pHxB2-MCS-Δ-env vector [16], [32]. For this study additional envs from chronic infection were cloned from viral RNA by nested reverse-transcriptase polymerase chain reaction (RT-PCR). Viral RNA was extracted from plasma (200 µl) using the QIAamp Viral RNA Kit (Qiagen UK), DNAse I treated (37°C, 1 hr) and a 10^th^ of the RNA was then RT (50°C 30 mins, 60°C 30 mins) and amplified using the Titan One Tube RT-PCR Kit (Roche Diagnostics GmbH, Germany) with forward primers 988L+ (5′-GTAGCATTAGCGGCCGCAATAATAATAGCAATAG-3′) and 943S+ (5′-CAATAGYAGCATTAGTAGTAG-3′) and either reverse primer 609RE- (5′-CCCATAGTGCTTCCGGCCGCTCCCAAG-3′) or 628L- (5′-TCATCTAGAGATTTATTACTCC-3′). For the nested PCR the Expand Long Template PCR System (Diagnostics GmbH, Germany) was employed following the reaction conditions specified by the manufacturer for buffer 3 and a 10^th^ of the first round product as template with primers 626L+ (5′-GTGGGTCACCGTCTATTATGGG-3′) and 125Y-(5′-CACCACGCGTCTCTTTGCCTTGGTGGG-3′), containing BstEII and MluI sites (underlined). The PCR conditions were: 30 cycles of 92°C 45s, 45 or 50°C for 45s and 68°C for 210s, finishing with a 10 mins elongation step at 68°C. The amplified env-fragments were cloned into pCR-TOPO (Promega, UK) and subsequently transferred into pHxB2-MCS-Δ-env, following digestion with BstEII and MluI. Plasmid pHxB2-MCS-Δ-env allows incorporation of heterologous env sequences from seven amino acids after the signal peptide to six amino acids prior to the gp120/gp41 junction [43]. Viruses were produced by transfection of 293T cells using FuGENE-HD (Roche Diagnostics, Indianapolis, USA).

### *Env* sequencing and phylogenetic analysis

*Envs* were sequenced using the Big Dye Terminator Kit 3.1 (Applied Biosystems, USA) using 3.2 pmol of primer and 500 ng of plasmid. Both strands were sequenced to give a 2 - 4-fold redundancy. The sequences and their chromatograms were assembled into a contig using Sequencher software (Gene Codes Corp.; USA). The GenBank accession numbers for the *envs* are: MM1 AY295200-1-6, DQ425059-61; MM2 AY295211, DQ425062-66-68; MM4 AY295223-5-6, DQ425069-71, DQ875805-8; MM8 AY295233-5-7, DQ425072-4, DQ645378-84; MM23 DQ425077-82, DQ875809-10, GQ304511-7; MM27 DQ425083-8; MM28 DQ425089-92.

Cloned *env* sequences were manually aligned with 200 HIV-1 subtype B *env* gene sequences extracted from the Los Alamos National Laboratory HIV database using the software Se-Al [44]. Hyper-variable regions that could not be unambiguously aligned were excluded. A maximum-likelihood phylogenetic tree was constructed under the general time-reversible model of nucleotide substitution with proportion of invariable sites and gamma-distributed rate heterogeneity (GTR+I+G), using the program PAUP* version 4.0b10 [45]. The robustness of the tree topology was assessed by neighbour-joining bootstrap analysis with 1,000 replicates.

### Strain specific PCR

Strain specific primers for nested PCR were designed to amplify ∼500 bp segments of the *env* gene of MM23′s viruses. The PCRs were optimised on plasmids encoding strain A and B *envs*. For amplification of proviral DNA the Expand Long Template PCR System (Roche, UK) was employed using 300–600 ng of PBMC DNA as template in the first PCR reaction, and a 10^th^ of the first round product for nesting, with buffer 3 and with the following cycling conditions: 96°C 5 mins, then 30 cycles of 96°C 60 s, 60°C (outer PCR) or 55°C (inner PCR) 60 s, and 68°C 60 s, finishing with a 10 mins elongation step at 68°C. For amplification of viral RNA the Titan One Tube RT-PCR Kit (Roche, UK) was employed for the RT-step and the first amplification round using RNA extracted from 20 µl of plasma, and primers 988L+, 943S+ and 609RE- (*see* Amplification of gp120). A 10^th^ of the RT-PCR product was then nested with the inner strain specific primers following the same conditions as for proviral DNA. The strain identity of the PCR products was confirmed by cycle sequencing using Big Dye Terminator Kit 3.1 (Applied Biosystems, USA) with 3.2 pmol of primer and 25 ng of agarose gel-purified product.

### Virus titrations

Human glioma (NP-2/U87) cells expressing CD4 and a putative coreceptor (CCR3, CCR5 or CXCR4;) were kindly donated by H Hoshino (Gunma University School of Medicine, Japan; NP-2 cells [46], [47]) or obtained from CFAR (NIBSC, UK; U87 cells [46], [47]), respectively, and grown in DMEM (Invitrogen, UK) supplemented with 10% FCS, 1 µg/ml puromycin and 100 µg/ml G418. Ten-fold serial dilutions of viral stocks were incubated in triplicate for 2 hrs at 37°C on semiconfluent cell layers. The cells were then washed and cultured for 48 hrs. Infection was detected by p24-immunostaining, as detailed elsewhere [16], [32]. Briefly, fixed cells were incubated with anti-HIV-1 p24 monoclonal antibodies (ADP 365 and 366, NIBSC, UK; 1:40 dilution) followed by an anti-mouse Ig antibody conjugated to β-galactosidase (Southern Biotechnology Associates, USA; at 2.5 µg/ml). After incubation with X-Gal substrate at 37°C, infected cells appear blue and focus-forming units (FFU) are counted.

### Neutralization assay

Two hundred FFU of HIV-1 were incubated with 2 (or 3)-fold serially diluted patient serum (from 1∶10 or 1∶20 dilution) or MAbs or sCD4 from 15 µg/ml, in a volume of 100 µl of DMEM supplemented with 10% FCS, for 1 hr at 37°C. As negative controls, parallel assays were run without antibody or, for serum assays, with pooled HIV-1 seronegative human serum (PAA Laboratories, UK) at a 1:10 dilution. MAb 17b was assayed both in the presence and absence of sCD4 (at a concentration which reduced infection by 50%). When sCD4 was included, reduction in infection was calculated against virus incubated with sCD4 alone. Following the incubation, the antibody-virus cocktail was added to NP-2/CD4/CCR5 (or NP-2/CD4/CXCR4 for HIV-1_MN/IIIB_) cells seeded in 48-well plates. After 2 hrs incubation at 37°C, the cells were washed and then cultured for 48 hrs. Infection was measured by p24-immunostaining (*see* virus titrations). The percentage infection in the presence of antibody (i.e. patient serum or MAb) was calculated using the following formula: 100× [average FFU in the presence of antibody/average FFU in the control]. The threshold for a positive neutralization reaction with MAbs/sCD4 was set to ≥50% reduction of infection, with IC_50_ values being calculated using the XLFit4 software (ID Business Solutions, UK). For serum assays, we report the average reciprocal end-point dilution at which ≥90% reduction of infection was observed, as the neutralizing activity of sera that do not achieve reductions in infection above 90% is intrinsically variable between experiments [16] Each serum/MAb-virus combination was assayed in triplicate, at least twice.

### Single genome amplification and cloning of gp160

Full-length viral *envs* (gp160) were amplified by Single Genome Amplification (SGA) as described by Keele *et al*., 2008 [29]. Briefly, ∼10,000 viral RNA copies were extracted from plasma using the QIAamp Viral RNA Kit and reverse transcribed (50°C for 60 mins, 55°C for 60 mins) using (10 units/µl) Super-Script III (Invitrogen) in the supplied 1× RT buffer with 0.5 mM dNTPs, 5 mM DTT, 2 units/µl RNaseOut (Invitrogen) and 0.25 µM reverse primer Env3Out (5′-TTGCTACTTGTGATTGCTCCATGT-3′). Following reverse transcription the Super-Script was heat-inactivated (85°C for 10 mins), and the cDNA was RNaseH (2 units; Invitrogen) treated at 37°C for 30 mins. The cDNA was diluted to yield amplification in less than 30% of nested PCR reactions using High Fidelity Platinum *Taq* DNA polymerase (Invitrogen) and primers Env5out (5′-TAGAGCCCTGGAAGCATCCAGGAAG-3′) and Env3out (for outer PCR), followed by primers Env5in (5′- caccTTAGGCATCTCCTATGGCAGGAAGAAG-3′) and Env3in (5′-GTCTCGAGATACTGCTCCCACCC-3′) for the nested PCR. The PCR amplifications were carried out in the presence of 1× High Fidelity Platinum PCR buffer, 2 mM MgSO_4_, 0.2 mM dNTPs, 0.2 µM of each primer and 0.025 units/µl Platinum *Taq*. The PCR conditions were: 94°C for 2 mins, followed by 35 (outer PCR) or 45 (nested PCR) cycles of 94°C for 15 s, 55°C for 30 s and 68°C for 4 mins, finishing with a 10 mins elongation step at 68°C. All PCR products were sequenced and any sequence with evidence of mixed bases was excluded from further analysis. For virus production amplicons were cloned into pcDNA3.1/V5-His-TOPO-TA (Invitrogen) and then sequence confirmed (to exclude cloning introduced changes). Envelope pseudotyped viruses were produced in 293T cells by co-transfection with the pSG3Δenv plasmid [48] using FuGENE-HD (Roche Diagnostics). The GenBank accession numbers for the SGA *envs* are: JN034137-99.

### Sequence swapping and site directed mutagenesis (SDM)

Molecular determinants of neutralization sensitivity was sought by swapping envelope regions between neutralization sensitive and resistant clones using the conserved restriction enzyme sites Bgl II and PpuM I, by Env residues 273 (nt 7041) and 368 (nt 7326; HXB2 numbering), in combination with the BstE II and Mlu I sites incorporated in the primers used for gp120 amplification. This was supplemented by SDM with oligonucleotides encoding the desired mutations using *Pfx* DNA polymerase (Invitrogen) in reactions containing 1× *Pfx* buffer, 50 mM MgCl_2_, 1 mM dNTPs, forward and reverse primers at 1 µM, 200 ng plasmid DNA and 0.05 units/µl *Pfx.* The PCR conditions were: 94°C for 3 mins, followed by 20 cycles of 94°C for 30 s, 60°C for 30 s and 68°C for 12 mins, finishing with a 20 min elongation step at 68°C. The template DNA was digested by incubation with Dpn I at 37°C for 2 hours after which the mutated DNA was rescued by transformation of TOP10 E. *coli* cells (Invitrogen). Both the swaps and the SDM was conducted in the pGEM-T easy vector and verified by sequencing before the mutated *envs* were transferred into the pHxB2-MCS-Δ-env for virus production and re-confirmed by sequencing.

### Macrophage infectivity assay

PBMC were prepared from HIV seronegative donors by density-gradient centrifugation (Lymphoprep, Axis-Shield). Monocyte-derived macrophages (MDM) were prepared by adherence as described previously [49], except that cells were harvested and replated at 2×10^6^ cells/ml following the initial overnight incubation, and left to differentiate for 7–14 days in RPMI 1640 supplemented with 20% autologous human serum and 20 ng/ml macrophage colony stimulating factor (R&D Systems, UK). Once differentiated, MDM in 48-well trays (4×10^5^ cells/well) were infected with 500-5,000 FFU viral stock (titred on NP-2 cells) in 200 µl RPMI 1640/20% autologous serum. Media was replaced after 24 hrs and virus production was detected after four days following intracellular p24 staining (*see* virus titrations).

## Supporting Information

Figure S1 **Amino acid sequence alignments of Envs amplified by traditional PCR and by SGA.** The figure show amino acid alignments of MM4 day 493 (A) and MM8 day 608 (B) Envs, with clones derived by single genome amplification (SGA) labelled accordingly (4.10_SGA1-34; 8.8_SGA1-21). The neutralization phenotype of tested Env clones is indicated by ‘-S’ for sensitive and ‘-R’ for resistant following the clone name. The numbering of amino acid residues is according to HIV-1_HXB2_ and for clarity only the region of *env* that is cloned into the pHxB2-MCS-Δ-*env* vector is shown (Env residues 35–504). Dashes (−) denote sequence identity, while dots (.) represent gaps introduced to optimise alignments. The hypervariable domains (V1, V2, V3, V4 and V5) are indicated above the alignments and shaded grey. All but two of the SGA-derived Envs are unique (MM8 SGA clones 16 and 17 were identical). The location of the restriction enzyme sites used for mapping is indicated above the alignments (and bolded), and also the residues changed by site directed mutation.
(DOCX)

Table S1 **Seroconversion data.** *^a^* The presence of anti-HIV antibodies was evaluated using four commercial assays following the manufacturer's instructions; Murex HIV-1.2.O (Abbott/murex), Wellcozyme HIV Recombinant VK 56/57 (Abbott/murex), Serovida HIV-1/2 (Fujirebio) and VIDAS HIV Duo (bioMérieux). Numbers refers to days post onset of PHI symptoms. *^b^* Serum samples were considered negative if no reactivity were detected in any of the assays, or if only VIDAS HIV Duo scored positive (detects both p24 antigens and anti-HIV antibodies). *^c^* Proviral DNA detectable by nested PCR. *^d^* A fully positive serology refers to a positive score in all four assays, including a positive score at a reciprocal serum dilution>256 in the Serovida HIV-1/2 assay. * Infection likely to have occurred within a three months (MM4 and MM8) and one month (MM23) period, respectively, with the time-point of the last possible exposure being indicated.
(DOCX)

Table S2 **Patient details – Viral Load, CD4 counts and** ***env*** **PCR.** *^a^* Days counted from onset of symptoms characteristic of primary HIV infection (PHI) illness. *^b^* VL, plasma viral load (RNA copies/ml) determined using Chiron 3.0 (Emeryville, Cal., USA). *^c^* CD4, CD4 cell numbers (cells/µl). *^d^* nd, not determined. *^e^* Source material for *env* PCR: DNA = PBMC proviral DNA, RNA = plasma viral RNA.
(DOC)
