# Supplementary material for: In Vivo Emergence of HIV-1 Highly Sensitive to Neutralizing Antibodies
Source: PLoS One. 2011 Aug 24;6(8):e23961. doi: 10.1371/journal.pone.0023961 (PMC3161086; doi:10.1371/journal.pone.0023961)
Supplement: Figure S1 — Amino acid sequence alignments of Envs amplified by traditional PCR and by SGA. The figure show amino acid alignments of MM4 day 493 (A) and MM8 day 608 (B) Envs, with clones derived by single genome amplification (SGA) labelled accordingly (4.10_SGA1-34; 8.8_SGA1-21). The neutralization phenotype of tested Env clones is indicated by ‘-S’ for sensitive and ‘-R’ for resistant following the clone name. The numbering of amino acid residues is according to HIV-1HXB2 and for clarity only the region of env that is cloned into the pHxB2-MCS-Δ-env vector is shown (Env residues 35–504). Dashes (−) denote sequence identity, while dots (.) represent gaps introduced to optimise alignments. The hypervariable domains (V1, V2, V3, V4 and V5) are indicated above the alignments and shaded grey. All but two of the SGA-derived Envs are unique (MM8 SGA clones 16 and 17 were identical). The location of the restriction enzyme sites used for mapping is indicated above the alignments (and bolded), and also the residues changed by site directed mutation. (DOCX) [file pone.0023961.s001.docx]

Supplementary Figure 1A: Amino acid alignment of MM4 day 493 Env sequences

**↓BstE II (36) V1/V2**

Consensus W**V**TVYYGVPV WKEATTTLFC ASDAKAYDTE VHNVWATHAC VPTDPNPQEV VLENVTENFN MWKNNMVEQM HEDVISLWDQ SLKPCVKLTP LCVTLNCTNN VTIINGTNAN NNTTNGTS?t

4.10.1-S ---------- ---------- ---------- ---------- ---------- ---------- ---------- ---------- ---------- ---------- ------I--- --------..

4.10.3-S ---------- ---------- ---------- ---------- ---------- ---------- ---------- ---------- ---------- ---------- ------I--- --------..

4.10_SGA24 ---------- ---------- ---------- ---------- ---------- ---------- ---------- ---------- ---------- ---------- ---------- --------..

4.10_SGA23 ---------- ---------- ---------- ---------- ---------- ---------- ---------- ---------- ---------- ---------- ---------- --------.T

4.10_SGA6 ---------- ---------- ---------- ---------- ---------- ---------- ---------- ---------- ---------- ---------- ---------- ------A...

4.10_SGA22 ---------- ---------- ---------- ---------- ---------- ---------- ---------- ---------- ---------- ---------- ---------- --------.T

4.10_SGA26 ---------- ---------- ---------- ---------- ---------- ---------- ---------- ---------- ---------- ---------- ---------- --------.T

4.10_SGA4 ---------- ---------- ---------- ---------- ---------- ---------- ---------- ---------- ---------- ---------- ---------- --..----..

4.10_SGA29-R ---------- ---------- ---------- ---------- ---------- ---------- ---------- ---------- ---------- ---------- -------S-- --------..

4.10_SGA15 ---------- ---------- ---------- ---------- ---------- ---------- ---------- ---------- ---------- ---------- ---------- --------..

4.10_SGA5 ---------- ---------- ---------- ---------- ---------- ---------- ---------- ---------- ---------- ---------- ---------- --------..

4.10_SGA1 ---------- ---------- ---------- ---------- ---------- G--------- ---------- ---------- ---------- -----H---- ---------- -------NAT

4.10_SGA18 ---------- ---------- ---------- ---------- ---------- ---------- ---------- ---------- ---------- -----H---- ---------- -------NAT

4.10_SGA34 ---------- ---------- ---------- ---------- ---------- ---------- ---------- ---------- ---------- -----H---- ---------- -------NAT

4.10_SGA8 ---------- ---------- ---------- ---------- ---------- ---------- ---------- ---------- ---------- -----H---- ---------- -------NAT

4.10_SGA9-S ---------- ---------- ---------- ---------- ---------- ---------- ---------- ---------- ---------- -----H---- ---------- -------NAI

4.10_SGA27 ---------- ---------- ---------- ---------- ---------- ---------- ---------- ---------- ---------- -----H---- ---------- -------NAT

4.10_SGA32 ---------- ---------- ---------- ---------- ---------- ---------- ---------- ---------- ---------- ---------- ---------- -------NAT

4.10_SGA33 ---------- ---------- ---------- ---------- ---------- ---------- ---------- ---------- ---------- ---------- ---------- -------NAT

4.10_SGA19 ---------- ---------- ---------- ---------- ---------- ---------- ---------- ---------- ---------- ---------- ---------- -------NAT

4.10_SGA20 ---------- ---------- ---------- ---------- ---------- ---------- ---------- ---------- ---------- ---------- ---------- ------AS.T

4.10_SGA21 ---------- ---------- ---------- ---------- ---------- ---------- ---------- ---------- ---------- ---------- ---------- ---I-D-NAT

4.10_SGA2 ---------- ---------- ---------- ---------- ---------- ---------- ---------- ---------- ---------- ---------- ------I--- --------..

4.10_SGA7 ---------- ---------- ---------- ---------- ---------- ---------- ---------- ---------- ---------- ---------- ------I--- --------..

4.10_SGA3 ---------- ---------- ---------- ---------- ---------- ---------- ---------- ---------- ---------- ---------- ---------- --------.T

4.10_SGA14 ---------- ---------- ---------- ---------- ---------- ---------- ---------- ---------- ---------- ---------- ---------- --------.T

4.10_SGA10 ---------- ---------- ---------- ---------- -----S---- ---------- ---------- ---------- ---------- ---------- -----S---- --N--D-NAT

4.10_SGA30 ---------- ---------- ---------- ---------- ---------- ---------- ---------- ---------- ---------- ---------- -----S---- --N--D-NAT

4.10_SGA12 ---------- ---------- ---------- ---------- ---------- ---------- ---------- ---------- ---------- ---------- ---------- --------AT

4.10_SGA16 ---------- -----P---- ---------- ---------- ---------- ---------- ---------- ---------- ---------- ---------- ---------- -------NAT

4.10_SGA31 ---------- ---------- ---------- ---------- ---------- ---------- ---------- ---------- ---------- -----H---- ---------- -------NAT

4.10_SGA17 ---------- ---------- ---------- ---------- ---------- ---------- ---------- ---------- ---------- ---------- ---------- -------NAT

4.10_SGA25 ---------- ---------- ---------- ---------- ---------- ---------- ---------- ---------- ---------- ---------- ---------- -------NAT

4.10_SGA28 ---------- ---------- ---------- ---------- ---------- ---------- ---------- ---------- ---------- ---------- ---------- --------.T

4.10_SGA11 ---------- ---------- ---------- ---------- ---------- ---------- ---------- ---------- ---------- ---------- ------I--- --------..

4.10_SGA13 ---------- ---------- ---------- ---------- ---------- ---------- ---------- ---------- ---------- ---------- ---------- --------.T

4.10.7-R ---------- ---------- ---------- ---------- ---------- ---------- ---------- --------V- ---------- ---------- ---------- --------.T

**V1/V2**

Consensus dannsswggi GRIDRGGEIK NCSFNITTSL SDKRQKEYAL FYKLDVEPID NDNTSYRLIS CNTSVITQAC PKVSFQPIPI HYCAPAGFAI LKCNDKKFNG TGPCKNVSTV QCTHGIRPVV

4.10.1-S .......... VK-------- ---------- ---------- ---------- ---------- ---------- ---------- ---------- ---------- ---------- ----------

4.10.3-S .......... VK-------- ---------- ---------- ---------- ---------- -------R-- -----R---- ---------- ---------- ---------- ----------

4.10_SGA24 .......... VK-------- ---------- ---------- ---------- ---------- ---------- ---------- ---------- ---------- ---------- ----------

4.10_SGA23 DANKSSWGEI ---------- ---------- ---------- ---------- ---------- ---------- ---------- ---------- ---------- ---------- ----------

4.10_SGA6 .....SWGRI ---------- ---------- ---------- ---------- ---------- ---------- ---------- ---------- ---------- ---------- ----------

4.10_SGA22 DANTSSWGGI ---------- ---------- ---------- ---------- ---------- ---------- ---------- ---------- ---------- ---------- ----------

4.10_SGA26 DANNSSWGEI ---------- ---------- ---------- ---------- ---------- ---------- ---------- ---------- ---------- ---------- ----------

4.10_SGA4 .......... VK-------- ---------- ---------- ---------- ---------- ---------- ---------- ---------- ---------- ---------- ----------

4.10_SGA29-R .......... VK-------- ---------- ---------- ---------- ---------- ---------- ---------- ---------- ---------- ---------- ----------

4.10_SGA15 .......... VK-------- ---------- ---------- ---------- ---------- ---------- ---------- ---------- ---------- ---------- ----------

4.10_SGA5 .......... VK-------- ---------- ---------- ---------- ---------- ---------- ---------- ---------- ---------- ---------- ----------

4.10_SGA1 DANNSSWEGI ---------- ---------- ---------- ---------N ---------- ---------- -----E---- ---------- ---------- ---------- ----------

4.10_SGA18 DANNSSWEGI ---------- ---------- ---------- ---------N ---------- ---------- ---------- ---------- ---------- ---------- ----------

4.10_SGA34 DANNSSWEGI ---------- ---------- ---------- ---------N ---------- ---------- ---------- ---------- ---------- ---------- ----------

4.10_SGA8 DANNSSWEGI ---------- ---------- ---------- ---------N ---------- ---------- -----E---- ---------- ---------- ---------- ----------

4.10_SGA9-S DANNSSWEGI ---------- ---------- ---------- ---------- ---------- ---------- ---------- ---------- ---------- ---------- ----------

4.10_SGA27 GANNSSWEGI ---------- ---------- ---------- ---------N ---------- ---------- -----E---- ---------- ---------- ---------- ----------

4.10_SGA32 DANNSSWGGI ---------- ---------- ---------- ---------- ---------- ---------- ---------- ---------- ---------- ---------- ----------

4.10_SGA33 DANNR..GGI ---------- ---------- ---------- ---------- ---------- ---------- ---------- ---------- ---------- ---------- ----------

4.10_SGA19 DANNSNWEGI ---------- ---------- ---------- ---------- ---------- ---------- ---T-E---- ---------- ---------- ---------- ----------

4.10_SGA20 DANNSSWGGI ---------- ---------- ---------- ---------- ---------- ---------- -----A---- ---------- ---------- ---------- ----------

4.10_SGA21 DANKSSWGGI ---------- ---------- ---------- ---------- ---------- ---------- ---------- ---------- ---------- ---------- ----------

4.10_SGA2 .......... VK-------- ---------- ---------- ---------- ---------- ---------- ---------- ---------- ---------- ---------- ----------

4.10_SGA7 .......... VK-------- ---------- ---------- ---------- ---------- ---------- ---------- ---------- ---------- ---------- ----------

4.10_SGA3 DANNSSWGEI ---------- ---------- ---------- ---------- ---------- ---------- ---------- ---------- ---------- ---------- ----------

4.10_SGA14 DANKSSWGGI ---------- ---------- ---------- ---------- ---------- ---------- ---------- ---------- ---------- ---------- ----------

4.10_SGA10 DANNSSWGGI ---------- ---------- ---------- ---------- ---------- ---------- ---------- ---------- ---------- ---------- ----------

4.10_SGA30 DANNSSWGGI ---------- ---------- ---------- ---------- ---------- ---------- ---------- ---------- ---------- ---------- ----------

4.10_SGA12 DANNSSWGGI ---------- ---------- ---------- ---------- ---------- ---------- ---------- ---------- ---------- ---------- ----------

4.10_SGA16 DANNSSWEGI ---------- ---------- ---------- ---------- ---------- ---------- ---------- ---------- ---------- ---------- ----------

4.10_SGA31 DANNSSWEGI ---------- ---------- ---------- ---------- ---------- ---------- ---------- ---------- ---------- ---------- ----------

4.10_SGA17 DANNNSWGGI ---------- ---------- ---------- ---------- ---------- ---------- ---------- ---------- ---------- ---------- ----------

4.10_SGA25 DANNNSWGGI ---------- ---------- ---------- ---------- ---------- ---------- ---------- ---------- ---------- ---------- ----------

4.10_SGA28 DANNSGWGGI ---------- ---------- ---------- ---------- ---------- ---------- ---T-E---- ---------- ---------- ---------- ----------

4.10_SGA11 .......... VK-------- ---------- ---------- ---------- ---------- ---------- ---------- ---------- ---------- ---------- ----------

4.10_SGA13 DANKSSWGGI ---------- ---------- ---------- ---------- ---------- ---------- ---------- ---------- ---------- ---------- ----------

4.10.7-R DANKSSWGGI ---------- ---------- ---------- ---------- ---------- ---------- ---------- ---------- ------E--- ---------- ----------

**↓Bgl II (273)** **V3 ↓328**

Consensus STQLLLNGSL AEEDIVI**R**SA NLTDNAKIII VQLNESVTIN CTRPNNNTRR GIHLGPGGAF ?TTGDIIGDI R**Q**AHCNISRA EWNKTLDRIA RELSKQFVNK TIVFKNSSGG **D**PEIVMHSFN

4.10.1-S ---------- ---------- ---------- ---------- ---------- ---------- Y--------- -**R**-------- ---------- -K--E--N-- ---------- ----------

4.10.3-S ---------- ---------- ---------- ---------- ---------- ---------- Y--------- -**R**-------- ---------- -K--E--N-- ---------- ----------

4.10_SGA24 ---------- ---------- -------T-- ---------- ---------- ---------- F--------- -**K**-------- ---------- -K--E--D-- ---------- ----------

4.10_SGA23 ---------- ---G------ ---------- ---------- ---------- ---------- Y--------- -**-**-------- ---------- -K-NE--N-- ---------- ----------

4.10_SGA6 ---------- -----I---- ---------- ---------- ---------- ---------- Y--------- -**-**-------- ---------- -E-------- --------E- ----------

4.10_SGA22 ---------- ---------- ---------- ---------- ---------- ---------- Y--------- -**-**-------- ---------- ---------- ---------- ----------

4.10_SGA26 ---------- ---------- ---------- ---------- ---------- ---------- F--------- -**-**-------- ---------- ---------- ---------- ----------

4.10_SGA4 ---------- ---------- ---------- ---------- ---------- ---------- Y--------- -**-**-------- ---------- ---------- ---------- ----------

4.10_SGA29-R ---------- ---------- ---------- ---------- ---------- ---------- Y--------- -**K**-------- ---------- ---------- ---------- ----------

4.10_SGA15 ---------- ---------- ---------- ---------- ---------- ---------- Y--------- -**-**-------- ---------- ---------- ---------- ----------

4.10_SGA5 ---------- ---------- ---------- ---------- ---------- ---------- F--------- -**-**-------- ---------- ---------- ---------- ----------

4.10_SGA1 ---------- ---------- ---------- ---------- ---------- ---------- F--------- -**-**-------- ---------- ---------- ---------- ----------

4.10_SGA18 ---------- ---------- ---N------ ---------- ---------- ---------- Y--------- -**-**-------- ---------- ---------- ---------- ----------

4.10_SGA34 ---------- ---G------ ---------- ---------- ---------- ---------- F--------- -**-**-------- ---------- TK-------- ---------- ----------

4.10_SGA8 ---------- ---------- ---------- ---------- ---------- ---------- F--------- -**-**-------- ---------- ---------- ---------- ----------

4.10_SGA9-S ---------- ---------- ---------- ---------- ---------- ---------- Y--------- -**K**-------- ---------- -K--E--N-- ---------- -------N--

4.10_SGA27 ---------- ---------- ---------- ---------- ---------- ---------- Y--------- -**-**-------- ---------- -K-NE--N-- ---------- ----------

4.10_SGA32 ---------- ---------- ---------- ---------- ---------- ---------- Y--------- -**-**-------- ---------- ---------- ---------- ----------

4.10_SGA33 ---------- ---------- ---------- ---------- ---------- ---------- F--------- -**-**-------- ---------- ---------- ---------- ----------

4.10_SGA19 ---------- ---G------ -------T-- ---------- ---------- ---------- F--------- -**-**-------- ---------- ---------- ---------- ----------

4.10_SGA20 ---------- ---G------ -------T-- ---------- ---------- ---------- F--------- -**-**-------- ---------- ---------- ---------- ----------

4.10_SGA21 ---------- ---------- -------T-- ---------- ---------- ---------- F--------- -**-**-------- ---------- ---------- ---------- ----------

4.10_SGA2 ---------- ---------- ---------- ---------- ---------- ---------- F--------- -**-**-------- ---------- TK-------- ----T----- ----------

4.10_SGA7 ---------- ---------- ---------- ---------- ---------- ---------- F--------- -**-**-------- ---------- TK-------- ----T----- ----------

4.10_SGA3 ---------- ---------- ---------- ---------- ---------- ---------- F--------- -**-**-------- ---------- TK-------- ---------- ----------

4.10_SGA14 ---------- ---------- ---------- ---------- ---------- ---------- F--------- -**-**-------- ---------- TK-------- ---------- ----------

4.10_SGA10 ---------- ------L--- ---N------ ---------- ---------- ---------- Y--------- -**-**-------- ---------- -K--N--D-- ---------- ----------

4.10_SGA30 ---------- ---------- ---N------ ---------- ---------- ---------- Y--------- -**-**-------- ---------- -K--N--D-- ---------- ----------

4.10_SGA12 ---------- ---------- ---------- ---------- ---------- ---------- F--------- -**-**-------- ---------- -K--E--D-- ---------- ----------

4.10_SGA16 ---------- ---------- ---N------ ---------- ---------- ---------- F--------- -**-**-------- ---------- -K--Q--D-- ---------- ----------

4.10_SGA31 ---------- ---------- ---N------ ---------- ---------- ---------- F--------- -**-**-------- ---------- ---------- ---------- ----------

4.10_SGA17 ---------- ---------- ---------- ---------- ---------- ---------- Y--------- -**-**-------- ---------- ---------- ---------- ----------

4.10_SGA25 ---------- ---G------ -------T-- ---------- ---------- ---------- F--------- -**-**-------- ---------- ---------- ---------- ---------.

4.10_SGA28 ---------- ---G------ ---N------ ---------- ---------- ---------- Y--------- -**-**-------- ---------- -K--N----- ---------- ----------

4.10_SGA11 ---------- ---------- ---------- ---------- ---------- ---------- Y--------- -**-**-------- ---------- -K-----D-- ---------- ----------

4.10_SGA13 ---------- ---------- ---------- ---------- ---------- ---------- Y--------- -**-**-------- ---------- ---------- ---------- ----------

4.10.7-R ---------- ---G------ -------T-- ---------- ---------- ---------- V--RA----- -**-**-----N-- ------E--V IK--E--N.- --A------- ----------

**V4 V5**

Consensus CRGEFFYCNS TPLFNSTWH? NGTLDSAGES SDNITLICRI KQFINLWQEV GKAMYAPPIR GQISCSSKIT GLLLTRDGG. IKNDTNKTEI FRPAGGDMRD NWRSELYKYK VVKIEPLGVA

4.10.1-S ---------- --------.. ---------- ---------- ---------- ---------- ---------- ---------. ---------- --------G- ---------- ----------

4.10.3-S ---------- --------.. ---------- ---------- ---------- ---------- ---------- ---------. ---------- ---------- ---------- ----------

4.10_SGA24 ---------- --------.. ---------- ---------- ---------- ---------- ---------- ---------N S--------- ---------- ---------- ----------

4.10_SGA23 ---------- ---------S ---------- ---------- ---------- ---------- ---------- ---------. ---------- ---------- ---------- ----------

4.10_SGA6 ---------- ---------P ---------- ---------- ---------- ---------- ---------- ---------. ---------- ---------- ---------- ----------

4.10_SGA22 ---------- ---------P ---------- ---------- ---------- ---------- ---------- ---------. ---------- ---------- ---------- ----------

4.10_SGA26 ---------- ---------P ---------- ---------- ---------- ---------- ---------- ---------. ---------- ---------- ---------- ----------

4.10_SGA4 ---------- ---------S ---------- ---------- ---------- ---------- ---------- ---------. ---------- ---------- ---------- ----------

4.10_SGA29-R ---------- ---------S ---------- ---------- ---------- ---------- ---------- ---------N S--------- ---------- ---------- ----------

4.10_SGA15 ---------- ---------S ---------- ------R--- ---------- ---------- ---------- ---------. ..-------- ---------- ---------- ----------

4.10_SGA5 ---------- ---------S ---------- ------K--- ---------- ---------- ---------- ---------T ---G------ ---------- ---------- ----------

4.10_SGA1 ---------- ---------P ---------- ---------- ---------- ---------- ---------- ---------. ..--N----- ---------- ---------- ----------

4.10_SGA18 ---------- ---------P ---------- ---------- ---------- ---------- ---------- ---------. ..--N----- ---------- ---------- ----------

4.10_SGA34 ---------- ---------P ---------- ---------- ---------- ---------- ---------- ---------. ..--N----- ---------- ---------- ----------

4.10_SGA8 ---------- ---------S ---------- ---------- ---------- ---------- ---------- ---------. ---------- ---------- ---------- ----------

4.10_SGA9-S ---------- --------.. ---------- ---------- ---------- ---------- ---------- ---------. ---------- ---------- ---------- ----------

4.10_SGA27 ---------- ---------S ---------- ---------- ---------- ---------- ---------- ---------. ---------- ---------- ---------- ----------

4.10_SGA32 ---------- ---------P ---------- ---V------ ---------- ---------- ---------- ---------. ..--N----- ---------- ---------- ----------

4.10_SGA33 ---------- ---------P ---------- ---------- ---------- ---------- ---------- ---------. ..--N----- ---------- ---------- ----------

4.10_SGA19 ---------- ---------S ---------- ---------- ---------- ---------- ---------- ---------. ---------- ---------- ---------- ----------

4.10_SGA20 ---------- ---------S ---------- ---------- ---------- ---------- ---------- ---------. ---------- ---------- ---------- ----------

4.10_SGA21 ---------- ---------S ---------- ---------- ---------- ---------- ---------- ---------. ---------- ---------- ---------- ----------

4.10_SGA2 ---------- ---------S ---------- ------T--- ---------- ---------- ---------- ---------. ---------- ---------- ---------- ----------

4.10_SGA7 ---------- ---------P ---------- ---------- ---------- ---------- ---------- ---------K SE-N--.L-- ---------- ---------- ----------

4.10_SGA3 ---------- ---------S ---------- ------R--- ---------- ---------- ---------- ---------T N--------- ---------- ---------- ----------

4.10_SGA14 ---------- ---------P ---------- ---------- ---------- ---------- ---------- ---------. ..--N----- ---------- ---------- ----------

4.10_SGA10 ---------- --------.. ---------- ---------- ---------- ---------- ---------- ---------. .----.SP-- ---------- ---------- ----------

4.10_SGA30 ---------- --------.. ----E----- ---------- ---------- ---------- ---------- ---------. .----.SP-- ---------- ---------- ----------

4.10_SGA12 ---------- --------.. ---------- ---------- ---------- ---------- ---------- ---------T .E----.H-- ---------- ---------- ----------

4.10_SGA16 ---------- ---------P ---------- ---------- ---------- ---------- ---------- ---------E -E---...-- ---------- ---------- ----------

4.10_SGA31 ---------- ---------S ---------- ---------- ---------- ---------- ---------- ---------E -E---...-- ---------- ---------- ----------

4.10_SGA17 ---------- --------RS -------E-- ---------- ---------- ---------- ---------- ---------E -E---...-- ---------- ---------- ----------

4.10_SGA25 .......... .......... .......... .......... ......---- ---------- ---------- ---------R -E...----- ---------- ---------- ----------

4.10_SGA28 ---------- ---------S ---------- ---------- ---------I ---------- ---------- ---------E -E---...-- ---------- ---------- ----------

4.10_SGA11 ---------- --------.. ---------- ------M--- ---------- ---------- -------N-- ---------. TE---Y-P-- ---------- ---------- ----------

4.10_SGA13 ---------- ---------P ---------- ---------- ---------- ---------- -------N-- ---------. TE---S.P-- ---------- ---------- ----------

4.10.7-R --------D- ---------P ----NGIG-V ------P--- ---------- ---------- -------N-- ---------. IE-G---P-- ---------- ---------- ----------

**Mlu I (503)**↓

Consensus PTTAK**R**R

4.10.1-S --K----

4.10.3-S --K----

4.10_SGA24 -------

4.10_SGA23 -------

4.10_SGA6 -------

4.10_SGA22 -------

4.10_SGA26 -------

4.10_SGA4 -------

4.10_SGA29-R -------

4.10_SGA15 -------

4.10_SGA5 -------

4.10_SGA1 -------

4.10_SGA18 -------

4.10_SGA34 -------

4.10_SGA8 -------

4.10_SGA9-S -------

4.10_SGA27 -------

4.10_SGA32 -------

4.10_SGA33 -------

4.10_SGA19 -------

4.10_SGA20 -------

4.10_SGA21 -------

4.10_SGA2 -------

4.10_SGA7 -------

4.10_SGA3 -------

4.10_SGA14 -------

4.10_SGA10 -------

4.10_SGA30 -------

4.10_SGA12 -------

4.10_SGA16 -------

4.10_SGA31 -------

4.10_SGA17 -------

4.10_SGA25 -------

4.10_SGA28 -------

4.10_SGA11 -------

4.10_SGA13 -------

4.10.7-R --K----

Supplementary Figure 1B: Amino acid alignment MM8 day 608 Env sequences

**↓BstE II (36)** **V1/V2**

Consensus W**V**TVYYGVPV WKEATTTLFC ASDAKAYDTE AHNVWATHAC VPTDPNPQEV VLENVTENFN MWKNNMVEQM HEDIISLWDQ SLKPCVKLTP LCVTLNCTNV KNVTNANSTS SNISSWERME

8.8.4-R ---------- ---------- ---------- ---------- ---------- ---------- ---------- ---------- ---------- ---------- ---------- ----------

8.8.8-S ---------- ---------- ---------- ---------- ---------- ---------- ---------- ---------- ---------- ---------- ---------- ----------

8.8.3-S ---------- ---------- ---------- ---------- ---------- ---------- ---------- ---------- ---------- ---------- ---------- ----------

8.8_SGA9 ---------- ---------- ---------- ---------- ---------- ---------- ---------- ---------- ---------- ---------- ---------- ----------

8.8_SGA13 ---------- ---------- ---------- ---------- ---------- ---------- ---------- ---------- ---------- ---------- ---------- ----------

8.8_SGA3 ---------- ---------- ---------- ---------- ---------- ---------- ---------- ---------- ---------- ---------- ---------- ----------

8.8_SGA14-R ---------- ---------- ---------- ---------- ---------- ---------- ---------- ---------- ---------- ---------- ---------- ----------

8.8_SGA11-S ---------- ---T------ ---------- ---------- ---------- ---------- ---------- ---------- ---------- ---------- ---------- ----------

8.8_SGA7 ---------- ---------- ---------- ---------- ---------- ---------- ---------- ---------- ---------- ---------- ---------- ----------

8.8_SGA1-R ---------- ---------- ---------- ---------- ---------- ---------- ---------- ---------- ---------- ---------- ---------- ---------G

8.8_SGA8 ---------- ---------- ---------- ---------- ---------- ---------- ---------- ---------- ---------- ---------- --A------- ----------

8.8_SGA5 ---------- ---------- ---------- ---------- ---------- ---------- ---------- ---------- ---------- ---------- ---------- ----------

8.8_SGA4 ---------- ---------- ---------- ---------- ---------- ---------- ---------- ---------- ---------- ---------- ---------- ----------

8.8_SGA10 ---------- ---------- ---------- ---------- ---------- ---------- ---------- ---------- ---------- ---------- ---------- ----------

8.8_SGA6 ---------- ---------- ---------- ---------- ---------- ---------- ---------- ---------- ---------- ---------- ---------- ----------

8.8_SGA18 ---------- ---------- ---------- ---------- ---------- ---------- ---------- ---------- ---------- ---------- ---------- A---------

8.8_SGA19 ---------- ---------- ---------- ---------- ---------- ---------- ---------- ---------- ---------- ---------- ---------- ----------

8.8_SGA15 ---------- ---------- ---------- ---------- ---------- ---------- ---------- ---------- ---------- ---------- ---------- ----------

8.8_SGA12 ---------- ---------- ---------- ---------- ---------- ---------- ---------- ---------- ---------- ---------- ---------- ----------

8.8_SGA21 ---------- ---------- ---------- ---------- ---------- ---------- ---------- ---------- ---------- ---------- ---------- ----------

8.8_SGA16 ---------- ---------- ---------- ---------- ---------- ---------- ---------- ---------- ---------- ---------- --A------- F-N---G---

8.8_SGA17 ---------- ---------- ---------- ---------- ---------- ---------- ---------- ---------- ---------- ---------- --A------- F-N---G---

8.8_SGA2 ---------- ---T------ ---------- ---------- ---------- ---------- ---------- ---------- ---------- ---------- --A------- F-N---GK--

8.8_SGA20 ---------- ---------- ---------- ---------- ---------- ---------- ---------- ---------- ---------- ---------- ---------- ----------

**V1/V2**

Consensus EGEIKNCSFN VTSIGNKYHK EYALLYKLDL VPIDK..NDT TSYTLINCNT SVITQACPKV SFEPIPIHYC APAGFAILQC NDKNFTGKGP CKNVSTVQCT HGIKPVVSTQ LLLNGSLAEE

8.8.4-R K--------- I--R---RL- ----F----- -S--N..--- ---------- ---------- ---------- ---------- ---------- ---------- ---------- ----------

8.8.8-S ---------- ---R---KLQ ----F----- -S--N..--- ---------- ---------- ---------- ---------- ---------- ---------- ---------- ----------

8.8.3-S ---------- ---------- ------R--- -----..--- ---------- ---------- ---------- ---------- ---------- -N-------- ---------- ----------

8.8_SGA9 ---------- ---------- ---------- -----..--- ---------- ---------I ---------- ---------- ---------- -N-------- ---------- ----------

8.8_SGA13 ---------- ---------- ---------- ----...--- ---------- ---------- ---------- ---------- ---------- -N-------- ---------- ----------

8.8_SGA3 ---------- ---------- ---------- -----..--- ---------- ---------- ---------- ---------- ---------- ---------- ---------- ----------

8.8_SGA14-R ---------- ---------- ---------- -----..--- ---------- ---------- ---------- ---------- ---------- ---------- ---------- ----------

8.8_SGA11-S ---------- ---------- ---------- ----N..--- ---------- ---------- ---------- ---------- ---------- ---------- ---------- ----------

8.8_SGA7 ---------- ---------- ---------- -----..--- ---------- ---------- ---------- ---------- ---------- ---------- ---------- ----------

8.8_SGA1-R ---------- ---------- ---------- -----..--- ---------- ---------I ---------- ---------- ---------- ---------- ---------- ----------

8.8_SGA8 ---------- ---------- ---------- ----NNN--- ---------- ---------- ---------- ---------- ---------- ---------- ---------- ----------

8.8_SGA5 ---------- ---------- ---IF----- --T--..--- ---------- ---------- ---------- ---------- ---------- -N-------- ---------- ----------

8.8_SGA4 ---------- ---------- ---------- -----..--- ---------- ---------- ---------- ---------- ---------- -N-------- ---------- ----------

8.8_SGA10 ---------- ---------- ---------- ----NNN--- ---------- ---------- ---------- ---------- ---------- -N-------- ---------- ----------

8.8_SGA6 ---------- -----K---- ---------- -----..--- ---------- ---------- ---------- ---------- ---------- -N-------- ---------- ----------

8.8_SGA18 ---------- ---------- ---------- -----..--- ---------- ---------- ---------- ---------- ---------- -N-------- ---------- ----------

8.8_SGA19 ---------- ---------- ---------- -----..--- ---------- ---------- ---------- ---------- ---------- -N-------- ---------- ----------

8.8_SGA15 ---------- ---------- ---------- -----..--- ---------- ---------- ---------- ---------- ---------- ---------- ---------- ----------

8.8_SGA12 ---------- -----K---- ----F----V ----.....N A--M------ ---------- ---------- ---------- ---------- -N-------- ---------- ----------

8.8_SGA21 ---------- ---------- ---------- -----..--N ---------- ---------- ---------- ---------- ---------- ---------- ---------- ------I---

8.8_SGA16 ---------- ---R---RL- ----C----- ----...--- ---------- ---------- ---------- ---------- ---------- ---------- ---------- ----------

8.8_SGA17 ---------- ---R---RL- ----C----- ----...--- ---------- ---------- ---------- ---------- ---------- ---------- ---------- ----------

8.8_SGA2 ---------- ---R---RL- ----F----- ----...--- ---------- ---------- ---------- ---------- ---------- ---------- ---------- ----------

8.8_SGA20 ---------- ---R---RL- ----F----- ----...--- ---------- ---------- ---------- ---------- ---------- -N-------- ---------- ----------

**V3 317 ↓PpuM I(368) V4**

Consensus GVVIRSENIT DNAKTIIVQL KDSVKINCTR PNNNTRRSIH LGPGKT**L**YAT DIIGDIRQAH CNISVADWNN TLQQIVIKLK EQFKNKTIIF NQSSGG**D**PEI VMHTFNCGGE FFYCNSTQLF

8.8.4-R --------F- ---------- ---------- ---------- ------**W**--- ---------- ---------- ---------G ---------- ---------- ---------- ----------

8.8.8-S ---------- ---------- ---------- ---------- ------**-**--- ---------- ---------- ---------- ---Q------ ---------- ---------- ----------

8.8.3-S ---------- ---------- ---------- ---------- ------**-**--- ---------- ---------- ---------- ---Q------ ---------- ---------- ----------

8.8_SGA9 ---------- ---------- ---------- ---------- ------**-**--- ---------- ---------- ---------- ---Q------ ---------- ---------- ----------

8.8_SGA13 ---------- ---------- ---------- ---------- ------**W**--- ---------- ---------- ---------- ---Q------ ---------- ---------- ----------

8.8_SGA3 ---------- ---------- ---------- ---------- ------**-**--- ---------- ----A----- ---------- ---Q------ ---------- ---------- ----------

8.8_SGA14-R ---------- ---------- ---------- ---------- ------**-**--- -------R-- ---------- ---------- ---Q------ ---------- ---------- ----------

8.8_SGA11-S ---------- ---------- ---------- ---------- ------**-**--- ---------- ----G----- ---------G ---------- ---------- ---------- ----------

8.8_SGA7 ---------- ---------- ---------- ---------- ------**-**--- ---------- ---------- ---------- ---Q------ ---------- ---------- ----------

8.8_SGA1-R --------F- ---------- ---------- ---------- ------**-**--- ---------- ---------- ---------- ---Q------ ---------- ---------- ----------

8.8_SGA8 ---------- ---------- ---------- ------K--- ------**W**--- ---------- ---------- ---------- ---Q------ KP-------- -T-------- ----------

8.8_SGA5 ---------- ---------- ---------- ------K--- ------**W**--- ---------- ---------- ---------G ---------- ---------- ---------- ----------

8.8_SGA4 --------F- ---------- ---------- ---------- ------**W**--- ---------- -----T---- ---------- ---------- KP-------- ---------- ----------

8.8_SGA10 --------F- ---------- ---------- ---------- ------**W**--- ---------- -----T---- ------K--- ---------- KP-------- ---------- ----------

8.8_SGA6 --------F- ---------- ---------- ---------- ------**W**--- ----N----- -----T---- ---------- ---------- ---------- ---------- ----------

8.8_SGA18 --------F- ---------- ---------- ---------- ------**W**--- ----N----- -----T---- ---------- ---------- ---------- ---------- ----------

8.8_SGA19 ---------- ---------- ---------- ---------- ------**W**--- ----N----- -----T---- ---------- ---Q------ ---------- ---------- ----------

8.8_SGA15 --------F- ---------- ---------- ---------- ------**W**--- ---------- ---------- ---------G ---------- ---------- ---------- ----------

8.8_SGA12 ---------- ---------- ---------- ---------- ------**-**--- ---------- ---------- ---------G ---------- ---------- ---------- ----------

8.8_SGA21 ---------- ---------- ---------- ---------- ------**W**--- ---------- ---------- ---------G ---------- ---------- ---------- ----------

8.8_SGA16 ---------- ---------- ---------- ------K--- ------**-**--- ----N----- ---------- ---------G ---------- ---------- ---------- ----------

8.8_SGA17 ---------- ---------- ---------- ------K--- ------**-**--- ----N----- ---------- ---------G ---------- ---------- ---------- ----------

8.8_SGA2 ---------- ---------- ---------- ------K--- ------**-**--- ---------- ---------- ---------G ---------- ---------- ---------- ----------

8.8_SGA20 ---------- ---------- ---------- ------K--- ------**-**--- ---------- ---------- ---------G ---------- ---------- ---------- ----------

**V4 399 V5 Mlu I (503)↓**

Consensus NDTWING**?**IQ SNST.ENDTI TLPCRIKQII NRWQEVGKAM YAPPIRGIIR CTSNITGLLL TRDGGNKNGS NN.NETFRPG GGDMRDNWRS ELYKYKVVKI EPLGVAPTKA K**R**R

8.8.4-R -------**T**-- ---------- ---------- ---------- ---------- ---------- ---------- ---------- ---------- ---------- ---------- ---

8.8.8-S -S-----**I**-- ---------- ---------- -----A---- ---------- ---------- ---------- ---------- ---------- ---------- ---------- ---

8.8.3-S -S-----**I**-- --I-T-..-- I--------- ---------- ---------- ---------- ---------- ---------- ---------- ---------- ---------- ---

8.8_SGA9 -S-----**I**-- --I-T-..-- I--------- ---------- ---------- ---------- ---------- ---------- ---------- ---------- ---------- ---

8.8_SGA13 -S-----**T**-P .-I-A-..-- I--------- ---------- ---------- ---------- ---------- ---------- ---------- ---------- ---------- ---

8.8_SGA3 -S-----**I**-- --I-A-..-- I--------- ---------- ---------- ---------- -----KN--- ---------- ---------- ---------- ---------- ---

8.8_SGA14-R -S-----**I**-- --I-A---N- ---------- ---------- ---------- ---------- ---------- ---------- ---------- ---------- ---------- ---

8.8_SGA11-S -S-----**I**-- --I-A-..-- I--------- ---------- ---------- ---------- --------E- ---T------ ---------- ---------- ---------- ---

8.8_SGA7 -S-----**I**-- ----A-..-- I--------- ---------- ---------- ---------- ---------- ---------- ---------- ---------- ---------- ---

8.8_SGA1-R -N-----**I**-- -------N-- I--------- ---------- ---------- ---------- ---------- S--T------ ---------- ---------- ---------- ---

8.8_SGA8 -S-----**N**-- --------N- ---------- ---------- ---------- ---------- ---------- ---------- ---------- ---------- ---------- ---

8.8_SGA5 -S-----**T**-P ---------- I--------- ---------- ---------- ---------- ---------- ---------- ---------- ---------- ---------- ---

8.8_SGA4 -------**T**-- -------EI- ---------- ---------- -------K-- ---------- ---------- ---------- ---------- ---------- ---------- ---

8.8_SGA10 -------**N**-- ---------- ---------- ---------- ---------- ---------- ---------- ---------- ---------- ---------- ---------- ---

8.8_SGA6 -------**A**-- ---------- ---------- ---------- ---------- ---------- ---------- --TI------ ---------- ---------- ---------- ---

8.8_SGA18 -------**T**-- ---------- ---------- ---------- ---------- ---------- -----KN--- ---------- ---------- ---------- ---------- ---

8.8_SGA19 -------**N**-- ---------- ---------- ---------- ---------- ---------- ---------- --TT------ ---------- ---------- ---------- ---

8.8_SGA15 -------**N**-- ---------- ---------- ---------- ---------- ---------- ---------- --TT------ ---------- ---------- ---------- ---

8.8_SGA12 -------**I**-- --------I- ---------- ---------- ---------- ---------- ---------- --TI------ ---------- ---------- ---------- ---

8.8_SGA21 -------**T**-- -------EI- ---------- ---------- ---------- ---------- ---------- --TT------ ---------- ---------- ---------- ---

8.8_SGA16 -------**T**-- ---------- ---------- ---------- ---------- ---------- -----KN-E- -.TT------ ---------- ---------- ---------- ---

8.8_SGA17 -------**T**-- ---------- ---------- ---------- ---------- ---------- -----KN-E- -.TT------ ---------- ---------- ---------- ---

8.8_SGA2 -------**T**-- ---------- ---------- ---------- -----K-Q-- -S-D------ -----KN-E- -.TT------ ---------- ---------- ---------- ---

8.8_SGA20 -------**T**-- ---.-..... ---------- ---------- -----K-Q-- -S-D------ -----KN-E- -.TT------ ---------- ---------- ---------- ---
