# Supplementary material for: In Vivo Emergence of HIV-1 Highly Sensitive to Neutralizing Antibodies
Source: PLoS One. 2011 Aug 24;6(8):e23961. doi: 10.1371/journal.pone.0023961 (PMC3161086; doi:10.1371/journal.pone.0023961)
Supplement: Table S1 — Seroconversion data. a The presence of anti-HIV antibodies was evaluated using four commercial assays following the manufacturer's instructions; Murex HIV-1.2.O (Abbott/murex), Wellcozyme HIV Recombinant VK 56/57 (Abbott/murex), Serovida HIV-1/2 (Fujirebio) and VIDAS HIV Duo (bioMérieux). Numbers refers to days post onset of PHI symptoms. b Serum samples were considered negative if no reactivity were detected in any of the assays, or if only VIDAS HIV Duo scored positive (detects both p24 antigens and anti-HIV antibodies). c Proviral DNA detectable by nested PCR. d A fully positive serology refers to a positive score in all four assays, including a positive score at a reciprocal serum dilution>256 in the Serovida HIV-1/2 assay. * Infection likely to have occurred within a three months (MM4 and MM8) and one month (MM23) period, respectively, with the time-point of the last possible exposure being indicated. (DOCX) [file pone.0023961.s002.docx]

**Table S1: Seroconversion data*^a^***

|  |  |  |  |  |  |  |
| --- | --- | --- | --- | --- | --- | --- |
| **Patient** | **Likely exposure** | **Last negative serology** *^b^* | **First diagnosis**  **(proviral DNA+)** *^c^* | **First positive serology** | **Fully positive serology** *^d^* | **First *env* cloning** |
|  |  |  |  |  |  |  |
|  |  |  |  |  |  |  |
| MM1 | -22 | -10 | 14 | 14 | 28 | 28 |
|  |  |  |  |  |  |  |
| MM2 | -12 | 4 | 4 | 17 | 19 | 32 |
|  |  |  |  |  |  |  |
| MM4 | -7* | -59 | 17 | 17 | 17 | 17 |
|  |  |  |  |  |  |  |
| MM8 | -18* | N/A | 5 | 5 | 12 | 12 |
|  |  |  |  |  |  |  |
| MM23 | -2* | 2 | 2 | 5 | 9 | 15 |
|  |  |  |  |  |  |  |
| MM27 | -20 | -135 | 12 | 12 | 26 | 28 |
|  |  |  |  |  |  |  |
| MM28 | -4 | 7 | 6 | 9 | 20 | 6 |
|  |  |  |  |  |  |  |

*^a^* The presence of anti-HIV antibodies was evaluated using four commercial assays following the manufacturer’s instructions; Murex HIV-1.2.O (Abbott/murex), Wellcozyme HIV Recombinant VK 56/57 (Abbott/murex), Serovida HIV-1/2 (Fujirebio) and VIDAS HIV Duo (bioMérieux). Numbers refers to days post onset of PHI symptoms.

*^b^* Serum samples were considered negative if no reactivity were detected in any of the assays, or if only VIDAS HIV Duo scored positive (detects both p24 antigens and anti-HIV antibodies).

*^c^* Proviral DNA detectable by nested PCR.

*^d^* A fully positive serology refers to a positive score in all four assays, including a positive score at a reciprocal serum dilution >256 in the Serovida HIV-1/2 assay.

* Infection likely to have occurred within a three months (MM4 and MM8) and one month (MM23) period, respectively, with the time-point of the last possible exposure being indicated.
