# Supplementary material for: In Vivo Emergence of HIV-1 Highly Sensitive to Neutralizing Antibodies
Source: PLoS One. 2011 Aug 24;6(8):e23961. doi: 10.1371/journal.pone.0023961 (PMC3161086; doi:10.1371/journal.pone.0023961)
Supplement: Table S2 — Patient details – Viral Load, CD4 counts and env PCR. a Days counted from onset of symptoms characteristic of primary HIV infection (PHI) illness. b VL, plasma viral load (RNA copies/ml) determined using Chiron 3.0 (Emeryville, Cal., USA). c CD4, CD4 cell numbers (cells/µl). d nd, not determined. e Source material for env PCR: DNA = PBMC proviral DNA, RNA = plasma viral RNA. (DOC) [file pone.0023961.s003.doc]

**Table S2: Patient details – Viral Load, CD4 counts and *env* PCR**

|  |  |  |  |  |  |  |  |  |  |  |
| --- | --- | --- | --- | --- | --- | --- | --- | --- | --- | --- |
| **MM1** | day*^a^* | 28 | 48 | 84 | 195 | 284 | 494 | 833 | 938 | 1879 |
|  | VL*^b^* | 34,400 | 14,900 | 5,00 | 60,000 | 63,400 | 63,400 | 169,300 | 180,500 | 111,600 |
|  | CD4*^c^* | nd*^d^* | nd | 1,000 | nd | 880 | 660 | 830 | 770 | 690 |
|  | *env* PCR*^e^* | DNA |  | DNA |  |  |  | RNA |  |  |
|  |  |  |  |  |  |  |  |  |  |  |
| **MM2** | day | 32 | 77 | 113 | 155 | 291 | 484 | 606 | 690 |  |
|  | VL | 426,600 | 144,200 | 218,100 | 163,100 | 104,300 | 48,400 | 34,300 | 26,400 |  |
|  | CD4 | nd | 330 | 350 | 450 | 270 | 260 | 330 | 270 |  |
|  | *env* PCR | DNA |  |  |  |  | RNA |  | RNA |  |
|  |  |  |  |  |  |  |  |  |  |  |
| **MM4** | day | 17 | 52 | 108 | 206 | 297 | 493 | 574 | 833 |  |
|  | VL | 160,000 | 9,900 | 42,300 | 30,200 | 24,00 | 19,900 | 34,500 | 137,200 |  |
|  | CD4 | nd | 990 | 590 | 750 | 610 | 690 | 610 | 650 |  |
|  | *env* PCR | DNA |  |  |  |  | RNA |  | RNA |  |
|  |  |  |  |  |  |  |  |  |  |  |
| **MM8** | day | 12 | 32 | 49 | 81 | 333 | 608 | 810 | 957 |  |
|  | VL | 5,927,000 | nd | 454,100 | 41,900 | 44,500 | 41,800 | 105,200 | 154,800 |  |
|  | CD4 | 290 | nd | 610 | 350 | 420 | 260 | 240 | 90 |  |
|  | *env* PCR | DNA | DNA |  |  |  | RNA |  | RNA |  |
|  |  |  |  |  |  |  |  |  |  |  |
| **MM23** | day | 15 | 37 | 64 | 113 | 204 | 316 | 722 | 1065 | 1534 |
|  | VL | 8,871,700 | nd | 200,500 | 147,700 | 117,600 | 110,100 | 52,400 | 45,700 | 168,300 |
|  | CD4 | 410 | nd | 410 | 510 | 330 | 300 | 550 | 240 | 190 |
|  | env PCR | DNA |  |  |  |  | RNA |  | RNA | RNA |
|  |  |  |  |  |  |  |  |  |  |  |
| **MM27** | day | 28 | 39 | 109 | 202 | 299 | 466 | 585 | 941 |  |
|  | VL | 353,200 | 67,200 | 33,700 | 28,800 | 34,400 | 10,600 | 26,400 | 85,500 |  |
|  | CD4 | 560 | 690 | 640 | 550 | 550 | 660 | 580 | 500 |  |
|  | *env* PCR | DNA |  |  |  |  | RNA |  |  |  |
|  |  |  |  |  |  |  |  |  |  |  |
| **MM28** | day | 6 | 9 | 62 | 93 | 198 | 405 | 503 |  |  |
|  | VL | 4,337,100 | 4,180,300 | 7,000 | 9,500 | 5,600 | 38,600 | 9,800 |  |  |
|  | CD4 | 560 | 390 | 630 | 660 | 840 | 620 | 570 |  |  |
|  | *env* PCR | DNA |  |  |  |  | RNA |  |  |  |

*^a^* Days counted from onset of symptoms characteristic of primary HIV infection (PHI) illness.

*^b^* VL, plasma viral load (RNA copies/ml) determined using Chiron 3.0 (Emeryville, Cal., USA).

*^c^* CD4, CD4 cell numbers (cells/μl).

*^d^* nd, not determined.

*^e^* Source material for *env* PCR: DNA = PBMC proviral DNA, RNA = plasma viral RNA.
